# Validation list no. 225: valid publication of new names and new combinations effectively published outside the IJSEM

**DOI:** 10.1099/ijsem.0.006863

**Published:** 2025-09-30

**Authors:** Aharon Oren, Markus Göker

**Affiliations:** 1 The Institute of Life Sciences, The Hebrew University of Jerusalem, The Edmond J. Safra Campus, 9190401 Jerusalem, Israel; 2 Leibniz Institute DSMZ – German Collection of Microorganisms and Cell Cultures, Inhoffenstrasse 7B, 38124 Braunschweig, Germany

The purpose of this list is to publish in the *International Journal of Systematic and Evolutionary Microbiology* (*IJSEM*) those new names and new combinations that were effectively published outside the *IJSEM* as set forth in Rule 27 of the International Code of Nomenclature of Prokaryotes (ICNP; 2022 Revision) [1]. Authors and other individuals wishing to have new names and/or combinations included in future lists should send an electronic copy of the published paper to the *IJSEM* Editorial Office for confirmation that all of the other requirements for valid publication have been met. It is also a requirement of *IJSEM* and the ICNP that authors of new species, new subspecies and new combinations provide evidence that types are deposited in two publicly accessible culture collections in two different countries. It should be noted that the date of valid publication of these new names and combinations is the date of publication of this list, not the date of the original publication of the names and combinations. The authors of the new names and combinations are as given below.

Inclusion of a name on these lists validates the publication of the name and thereby makes it available in the nomenclature of prokaryotes. Inclusion of a name on these lists is part of the requirements of a valid publication of an effectively published name. Indeed, some of these names may, in time, be considered later synonyms, or it may be proposed to transfer the organisms to another genus, thus necessitating the creation of a new combination. Conversely, it may be proposed to transfer an organism back to an earlier genus and to regard the basonym or an earlier new combination as the correct name instead of a more recent new combination. The ICNP does not decide on such taxonomic opinions.

**Table:** 

Name/authors	Proposed as	Nomenclatural type^1^	Priority^2^	Reference
*Acinetobacter chenhuanii* He *et al*. 2025, 1^3,4^	sp. nov.	XH1741 (=GDMCC 1.4480=KCTC 8531)	160	[2]
*Acinetobacter yuyunsongii* He *et al*. 2025, 1^3,4^	sp. nov.	XH1639 (=GDMCC 1.4479=KCTC 8532)	160	[2]
*Actinopolyspora lacussalsi* subsp. *lacussalsi* (Guan *et al*. 2013) Saker *et al*. 2022, 9^3^	comb. nov. [basonym: *Actinopolyspora lacussalsi* Guan *et al*. 2013]	DSM 46731=KCTC 19657^5^	156	[3]
*Actinopolyspora lacussalsi* subsp. *righensis* (Meklat *et al*. 2014) Saker *et al*. 2022, 9^3^	comb. nov. [basonym: *Actinopolyspora righensis* Meklat *et al*. 2014]	H23 (=DSM 45501=CCUG 63368)^6^	156	[3]
*Aeromonas salmonicida* subsp. *oncorhynchi* Ajmi *et al*. 2025, 16^3^	subsp. nov.	A-9 (=DSM 117494=LMG 33538)	154	[4]
*Affinirhizobium* Ma *et al*. 2023, 9^3^	gen. nov.	*Affinirhizobium pseudoryzae*	157	[5]
*Affinirhizobium pseudoryzae* (Zhang *et al*. 2011) Ma *et al*. 2023, 12^3^	comb. nov. [basonym: *Rhizobium pseudoryzae* Zhang *et al*. 2011]^7^	J3-A127 (=DSM 19479=KCTC 23294)^8^	157	[5]
*Affinirhizobium rhizoryzae* (Zhang *et al*. 2014) Ma *et al*. 2023, 12^3^	comb. nov. [basonym: *Rhizobium rhizoryzae* Zhang *et al*. 2014]	J3-AN59 (=DSM 29514=KCTC 23652)^9^	157	[5]
*Akkermansia massiliensis* Ndongo *et al*. 2022, 5^3^	sp. nov.	Marseille-P6666 (=CECT 30548=CSUR P6666)	6	[6]
*Allorhizobium paknamense* (Kittiwongwattana and Thawai 2013) Mousavi *et al*. 2015, 88	comb. nov. [basonym: *Rhizobium paknamense* Kittiwongwattana and Thawai 2013]	L6-8 (=DSM 100301=NBRC 109338)	158	[7]
*Altererythrobacter arenosus* Ahn *et al*. 2025, 6^3^	sp. nov.	CAU 1644 (=KCTC 92082=MCCC 1K07086)	127	[8]
*Altererythrobacter segetis* Lee and Whang 2021, 394	sp. nov.	YJ20 (=KACC 19554=NBRC 113199)	145	[9]
*Alteromonas arenosi* Luo *et al*. 2024, 8^3^	sp. nov.	ASW11-36 (=KCTC 82496=MCCC 1K05585)	70	[10]
*Anaerocellum diazotrophicum* corrig. (Chen *et al*. 2021) Bing 2024, 167^10^	comb. nov. [basonym: *Caldicellulosiruptor diazotrophicus* Chen *et al*. 2021	YA01 (=DSM 112098=JCM 34253)	179	[11]
*Anaerofustis butyriciformans* Wang *et al*. 2025, 15^3^	sp. nov.	HA2171 (=CGMCC 1.18050=KCTC 25721)	7	[12]
*Anaerophilus* Zhang *et al*. 2020, 423	gen. nov.	*Anaerophilus nitritigenes* corrig.	45	[13]
*Anaerophilus nitritigenes* corrig. Zhang *et al*. 2020, 424^11^	sp. nov.	MJB2 (=KCTC 15800=MCCC 1K03631)	45	[13]
*Anaerorudis* El Houari *et al*. 2025, 12^3,12^	gen. nov.	*Anaerorudis cellulosivorans*	1	[14]
*Anaerorudis cellulosivorans* El Houari *et al*. 2025, 12^3,13^	sp. nov.	m5 (=ATCC TSD-267=DSM 112743)	1	[14]
*Ancylomarina psychrotolerans* Jia *et al*. 2018, 1188	sp. nov.	4SWWS2-6 (=KCTC 15504=MCCC 1K01618)	79	[15]
*Anditalea* Shi *et al*. 2012, 705	gen. nov.	*Anditalea andensis*	166	[16]
*Anditalea andensis* Shi *et al*. 2012, 707	sp. nov.	ANESC-S (=CICC 10485=NCCB 100412)	166	[16]
*Arachidicoccus ginsenosidimutans* Siddiqi *et al*. 2017, 46749^14^	sp. nov.	BS20 (=KACC 18624=LMG 29195)	163	[17]
*Arthrobacter dokdonensis* corrig. Koh *et al*. 2019, 736^15^	sp. nov.	DCT-5 (=KCTC 49189=LMG 31284)	117	[18]
*Ascidiaceibacter* Chen *et al*. 2018, 1683	gen. nov.	*Ascidiaceibacter salegens*	29	[19]
*Ascidiaceibacter salegens* Chen *et al*. 2018, 1683	sp. nov.	HQA918 (=KCTC 52719=MCCC 1K03259)	29	[19]
*Atopomonas sediminilitoris* Li *et al*. 2023, 105	sp. nov.	A3.4 (=LMG 32563=MCCC 1K07166)	116	[20]
*Bacillus phocaeensis* Cadoret *et al*. 2017, 57	sp. nov.	SIT16 (=CCUG 69739=CSUR P2184)	113	[21]
*Bifidobacterium hominis* Hitch *et al*. 2025, 11^3^	sp. nov.	CLA-AA-H311 (=DSM 118068=LMG 33596)^16^	28	[22]
*Bifidobacterium platyrrhinorum* Modesto *et al*. 2025, 2^3^	sp. nov.	SMA15 (=BCRC 81224=NBRC 114051)^17^	91	[23]
*Bifidobacterium saimiriisciurei* Modesto *et al*. 2025, 2^3^	sp. nov.	SMA1 (=BCRC 81223=NBRC 114049)^18^	91	[23]
*Blautia aquisgranensis* Hitch *et al*. 2025, 11^3^	sp. nov.	CLA-JM-H16 (=DSM 114586=LMG 33033)	28	[22]
*Blautia caccae* Hitch *et al*. 2025, 12^3^	sp. nov.	CLA-SR-H028 (=DSM 118556=LMG 33609)	28	[22]
*Blautia intestinihominis* Hitch *et al*. 2025, 12^3^	sp. nov.	CLA-AA-H95 (=DSM 111354=LMG 33582)	28	[22]
*Brevibacterium metallicum* corrig. Román-Ponce *et al*. 2015, 1154^19^	sp. nov.	NM2E3 (=HAMBI 3627=LMG 28673)^20^	64	[24]
*Brevundimonas vitis* corrig. Jiang *et al*. 2021, 11^3,21^	sp. nov.	GR-TSA-9 (=CGMCC 1.18820=KCTC 82386)	124	[25]
*Brumimicrobium oceani* Zhang *et al*. 2023, 1382	sp. nov.	C305 (=KCTC 62371=MCCC 1H00297)	89	[26]
*Burkholderia paludis* Ong *et al*. 2016, 11^3^	sp. nov.	MSh1 (=DSM 100703=MCCC 1K01245)	46	[27]
*Chitinibacter mangrovi* Xu *et al*. 2025, 8^3^	sp. nov.	FCG-7 (=GDMCC 1.4868=KCTC 8742)^22^	180	[28]
*Chitinophaga defluvii* Liu *et al*. 2025, 7^3^	sp. nov.	H8 (=CCTCC AB 2023228=KCTC 102174)	100	[29]
*Chryseobacterium gotjawalense* Kim *et al*. 2024, 5^3^	sp. nov.	wdc7 (=JCM 35602=KCTC 92440)	61	[30]
*Chryseobacterium tagetis* Chhetri *et al*. 2022, 318	sp. nov.	RG1 (=KCTC 82696=NBRC 115057)	87	[31]
*Chryseolinea soli* Lee *et al*. 2019, 125	sp. nov.	KIS68-18 (=KACC 17327=NBRC 113100)	146	[32]
*Chryseosolibacter* Octaviana *et al*. 2022, 1066	gen. nov.	*Chryseosolibacter histidinae* corrig.	75	[33]
*Chryseosolibacter histidinae* corrig. Octaviana *et al*. 2022, 1066^23^	sp. nov.	PWU4 (=DSM 111594=NCCB 100798)	75	[33]
*Chryseosolibacter indicum* corrig. Octaviana *et al*. 2022, 1067^24^	sp. nov.	PWU20 (=DSM 111597=NCCB 100800)	75	[33]
*Clostridium amazonitimonense* Alou *et al*. 2018, 137^25^	sp. nov.	LF2 (=CSUR P1445=DSM 28600)	118	[34]
*Clostridium brassicae* Wang *et al*. 2023, 5^3^	sp. nov.	ZC22-4 (=JCM 35370=MCCC 1K07510)	54	[35]
*Clostridium pacaense* Hosny *et al*. 2019, 9^3,26^	sp. nov.	Marseille-P3100 (=CCUG 71489=CSUR P3100)	119	[36]
*Cohnella caldifontis* Xiang *et al*. 2024, 7^3^	sp. nov.	YIM B05605 (=KCTC 43462=NBRC 115921)^27^	39	[37]
*Consotaella aegiceratis* Luo *et al*. 2025, 6^3,28^	sp. nov.	CSK11QG-6 (=JCM 35044=MCCC 1K07176)	148	[38]
*Coprococcus intestinihominis* Hitch *et al*. 2025, 12^3^	sp. nov.	CLA-AA-H190 (=DSM 114688=LMG 33015)	28	[22]
*Corynebacterium haematomassiliense* corrig. Boxberger *et al*. 2020, 6^3,29^	sp. nov.	Marseille-Q3615 (=CECT 30226=CSUR-Q3615)	129	[39]
*Cyclobacterium sediminis* Shin and Kahng 2017, 94	sp. nov.	SD70 (=JCM 30860=KCTC 42471)	142	[40]
*Cytobacillus mangrovibacter* Shi *et al*. 2025, 7^3^	sp. nov.	FJAT-53684 (=GDMCC 1.3071=JCM 35618)	105	[41]
*Cytobacillus spartinae* Shi *et al*. 2025, 7^3^	sp. nov.	FJAT-54145 (=GDMCC 1.3079=JCM 35621)	105	[41]
*Dawidia* Octaviana *et al*. 2022, 1069	gen. nov.	*Dawidia cretensis*	75	[33]
*Dawidia cretensis* Octaviana *et al*. 2022, 1069	sp. nov.	PWU5 (=DSM 111596=NCCB 100799)	75	[33]
*Dawidia soli* Octaviana *et al*. 2022, 1069	sp. nov.	PWU37 (=DSM 111595=NCCB 100801)	75	[33]
*Defluviimonas salinarum* Lee *et al*. 2023, 6^3^	sp. nov.	CAU 1641 (=KCTC 92081=MCCC 1K07180)	40	[42]
*Deinococcus guangxiensis* Sun *et al*. 2009, 922	sp. nov.	WGR700 (=CICC 10360=JCM 15082)^30^	171	[43]
*Deinococcus oregonensis* Gundlapally and Garcia-Pichel 2017, 74	sp. nov.	OR316-6 (=DSM 17762=JCM 13503)	30	[44]
*Desulfofustis limnaeus* Watanabe *et al*. 2022, 4^3,31^	sp. nov.	PPLL (=DSM 110475=JCM 39161)	66	[45]
*Desulfurivibrio dismutans* Sorokin *et al*. 2025, 11^3,32^	sp. nov.	AMeS2 (=DSM 113758=JCM 39203)^33^	133	[46]
*Dethiobacteraceae* Sorokin and Merkel 2022, 1^3^	fam. nov.	*Dethiobacter*	2	[47]
*Dethiobacterales* Sorokin and Merkel 2022, 1^3^	ord. nov.	*Dethiobacter*	3	[48]
*Dethiobacteria* Sorokin and Merkel 2022, 1^3^	class. nov.	*Dethiobacter*	4	[49]
*Devosia alba* Qiu *et al*. 2025, 6^3^	sp. nov.	RY10 (=KCTC 72161=MCCC 1K03748)	99	[50]
*Dokdonia ponticola* Park *et al*. 2018, 1130	sp. nov.	OISW-17 (=CGMCC 1.16527=KCTC 62187)^34^	25	[51]
*Dyadobacter luteus* Chen *et al*. 2020, 195	sp. nov.	RS19 (=CGMCC 1.13719=JCM 32940)^35^	71	[52]
*Echinicola arenosa* Baek *et al*. 2021, 5680	sp. nov.	CAU 1574 (=KCTC 82410=MCCC 1K05669)	86	[53]
*Edaphovirga* Xue *et al*. 2019, 340^36^	gen. nov.	*Edaphovirga cremea*	72	[54]
*Edaphovirga cremea* Xue *et al*. 2019, 341^37^	sp. nov.	SYP-B2100 (=DSM 105170=KCTC 62024)^38^	72	[54]
*Enterobacter intestinihominis* Hitch *et al*. 2025, 12^3^	sp. nov.	CLA-AC-H004 (=DSM 118557=LMG 33610)	28	[22]
*Enterocloster hominis* Hitch *et al*. 2025, 12^3^	sp. nov.	CLA-SR-H021 (=DSM 118482=LMG 33606)	28	[22]
*Entomoplasma multilocale* (Hill *et al*. 1992) Gasparich and Kuo 2019, 2737	comb. nov. [basonym: *Acholeplasma multilocale* Hill *et al*. 1992]^39^	PN525 (=ATCC 49900=DSM 116231)	14	[55]
*Erwinia aeris* Guo *et al*. 2025, 5^3^	sp. nov.	ACCC 02193 (=GDMCC 1.4563=JCM 37069)	98	[56]
*Faecalibacterium intestinale* Hitch *et al*. 2025, 12^3^	sp. nov.	CLA-AA-H281 (=DSM 116193=LMG 33027)	28	[22]
*Feifantangia* Zheng *et al*. 2015, 1446	gen. nov.	*Feifantangia zhejiangensis*	33	[57]
*Feifantangia zhejiangensis* Zheng *et al*. 2015, 1446	sp. nov.	NMD7 (=KCTC 42445=MCCC 1K00458)	33	[57]
*Fervidibacter* Nou *et al*. 2025, 2^3^	gen. nov.	*Fervidibacter sacchari*	19	[58]
*Fervidibacter sacchari* Nou *et al*. 2025, 2^3^	sp. nov.	PD1 (=JCM 39283=DSM 113467)	19	[58]
*Fervidibacteraceae* Nou *et al.* 2025, 2^3^	fam. nov.	*Fervidibacter*	19	[58]
*Fervidibacterales* Nou *et al*. 2025, 2^3^	ord. nov.	*Fervidibacter*	19	[58]
*Fervidibacteria* Nou *et al*. 2025, 2^3^	class. nov.	*Fervidibacter*	19	[58]
*Fictibacillus iocasae* Wang *et al*. 2018, 1127	sp. nov.	S38 (=CGMCC 1.16031=DSM 104298=KCTC 33865)	31	[59]
*Flagellimonas algarum* Yoon *et al*. 2025, 459	sp. nov.	DF-77 (=KCTC 72791=NBRC 114251)	77	[60]
*Flaviaestuariibacter flavus* corrig. Jang *et al*. 2020, 2667^40^	sp. nov.	17J68-12 (=JCM 33179=KCTC 62219)	88	[61]
*Flavisolibacter longurius* Maeng *et al*. 2021, 2827	sp. nov.	BT320 (=KCTC 72422=NBRC 114375)	114	[62]
*Flavobacterium algoriphilum* Yang *et al*. 2025, 8^3^	sp. nov.	LB3P122 (=CGMCC 1.11443=NBRC 114820)	143	[63]
*Flavobacterium algoris* Yang *et al*. 2025, 10^3^	sp. nov.	GB2R13 (=CGMCC 1.24741=NBRC 114830)	143	[63]
*Flavobacterium arabinosi* Yang *et al*. 2025, 8^3^	sp. nov.	LT1R49 (=CGMCC 1.11617=NBRC 114822)	143	[63]
*Flavobacterium cryoconiti* Yang *et al*. 2025, 8^3^	sp. nov.	ZT3R17 (=CGMCC 1.11707=NBRC 114824)	143	[63]
*Flavobacterium galactosi* Yang *et al*. 2025, 9^3^	sp. nov.	ZT3R25 (=CGMCC 1.11711=NBRC 114825)	143	[63]
*Flavobacterium ginsengiterrae* Kim *et al*. 2011, 345	sp. nov.	DCY55 (=JCM 17337=KCTC 23319)	169	[64]
*Flavobacterium melibiosi* Yang *et al*. 2025, 9^3^	sp. nov.	XS2P12 (=CGMCC 1.23198=NBRC 114826)	143	[63]
*Flavonifractor hominis* Hitch *et al*. 2025, 13^3^	sp. nov.	CLA-AP-H34 (=DSM 118484=LMG 33602)	28	[22]
*Frateuria hangzhouensis* Yang *et al*. 2025, 8^3^	sp. nov.	STR12 (=ACCC 61897=GDMCC 1.2964=JCM 35226)	107	[65]
*Gallaecimonas mangrovi* Zhang *et al*. 2018, 1861	sp. nov.	HK-28 (=KCTC 62177=MCCC 1K03441)	60	[66]
*Gallibacterium faecale* Ryu *et al*. 2025, 6^3^	sp. nov.	AGMB14963 (=KCTC 25487=NBRC 116419)	101	[67]
*Gracilimonas aurantiaca* Jayan *et al*. 2025, 7^3,41^	sp. nov.	MJW-20 (=JCM 37229=KCTC 102297)^42^	112	[68]
*Haloarcula montana* Liu *et al*. 2025, 10^3^	sp. nov.	GH36 (=CGMCC 1.62631=MCCC 4K00122)	155	[69]
*Haloimpatiens massiliensis* Anani *et al*. 2020, 3^3^	sp. nov.	Marseille-P1935 (=CSUR P1935=DSM 100591)	121	[70]
*Halomarinibacterium* Jeong *et al*. 2022, 6^3^	gen. nov.	*Halomarinibacterium sedimenti* ^43^	78	[71]
*Halomarinibacterium sedimenti* Jeong *et al*. 2022, 6^3,44^	sp. nov.	CAU 1614 (=KCTC 82457=MCCC 1K06083)	78	[71]
*Halomonas marinisediminis* Zhao *et al*. 2020, 3778	sp. nov.	204 (=KCTC 62957=MCCC 1H00366)	36	[72]
*Halomonas sulfidivorans* Wang and Shao 2021, 15^3^	sp. nov.	NLG_F1E (=KCTC 72091=MCCC 1A13718)	55	[73]
*Heminiphilus* Park *et al*. 2021, 280	gen. nov.	*Heminiphilus faecis*	83	[74]
*Heminiphilus faecis* Park *et al*. 2021, 284	sp. nov.	AM35 (=DSM 110151=KCTC 15907)	83	[74]
*Heterorhizobium* Ma *et al*. 2023, 8^3^	gen. nov.	*Heterorhizobium halophytocola*	157	[5]
*Heterorhizobium halophytocola* (Bibi *et al*. 2012) Ma *et al*. 2023, 10^3^	comb. nov. [basonym: *Rhizobium halophytocola* Bibi *et al*. 2012]	YC6881 (=DSM 21600=LMG 26977)^45^	157	[5]
*Holosporineae* (Szokoli *et al*. 2020) Castelli and Petroni 2025, 16^3^	subord. nov.	*Holospora*	136	[75]
*Hominiventricola aquisgranensis* Hitch *et al*. 2025, 13^3^	sp. nov.	CLA-AA-H78B (=DSM 111355=LMG 33583)	28	[22]
*Hymenobacter duratus* Damdintogtokh *et al*. 2021, 3339	sp. nov.	BT646 (=KCTC 21915=NBRC 114854)	44	[76]
*Hymenobacter lucidus* Oh *et al*. 2022, 4^3^	sp. nov.	BT178 (=KCTC 72333=NBRC 115442)	43	[77]
*Hyphomonas pacifica* Li *et al*. 2016, 1118	sp. nov.	T16B2 (=LMG 27911=MCCC 1A04387)	20	[78]
*Intrasporangium zincisolvens* Feng *et al*. 2025, 6^3^	sp. nov.	YIM S08009 (=CGMCC 1.60168=NBRC 116604)^46^	102	[79]
*Iocasia* Zhang *et al*. 2021, 9^3,47^	gen. nov.	*Iocasia frigidifontis* corrig.	115	[80]
*Iocasia frigidifontis* corrig. Zhang *et al*. 2021, 10^3,48^	sp. nov.	NS-1 (=KCTC 15988=MCCC 1K04439)	115	[80]
*Jiella avicenniae* Zhang *et al*. 2022, 7^3,49^	sp. nov.	CBK1P-4 (=CGMCC 1.18742=JCM 34330)	152	[81]
*Lachnoanaerobaculum sanguinis* Suenari *et al*. 2025, 5^3^	sp. nov.	TM49 (=DSM 116945=JCM 36186)	27	[82]
*Lachnospira rogosae* Hitch *et al*. 2025, 14^3^	sp. nov.	CLA-AA-H255 (=DSM 118602=LMG 33594)	28	[22]
*Lacunisphaera* Rast *et al*. 2017, 14^3,50^	gen. nov.	*Lacunisphaera limnophila*	130	[83]
*Lacunisphaera limnophila* Rast *et al*. 2017, 14^3,51^	sp. nov.	IG16b (=DSM 26815=LMG 29469)	130	[83]
*Lacunisphaera parvula* Rast *et al*. 2017, 14^3^	sp. nov.	IG15 (=DSM 26814=LMG 29468)	130	[83]
*Laedolimicola intestinihominis* Hitch *et al*. 2025, 14^3^	sp. nov.	CLA-AA-H132 (=DSM 117481=LMG 33588)	28	[22]
*Leifsonia flava* Cai *et al*. 2018, 553	sp. nov.	SYP-B2174 (=CGMCC 1.15856=DSM 105144)^52^	42	[84]
*Lysinibacillus zambalensis* Aja *et al*. 2025, 12^3^	sp. nov.	M3 (=BIO TECH 10973=TISTR 10640)	76	[85]
*Lysobacter tyrosinilyticus* corrig. Du *et al*. 2015, 369^53^	sp. nov.	THG-DN8.2 (=JCM 30320=KCTC 42235)	73	[86]
*Macrococcus capreoli* Schiffer and Ehrmann 2025, 6^3^	sp. nov.	TMW 2.2395 (=DSM 113939=LMG 32618)	153	[87]
*Macrococcus psychrotolerans* Mašlaňová *et al*. 2025, 12^3^	sp. nov.	NRL/St 95/376 (=CCM 8659=DSM 111350)	18	[88]
*Macromonas nakdongensis* Baek and Choi 2023, 9^3,54^	sp. nov.	BK-30 (=JCM 31376=KCTC 52161)^55^	37	[89]
*Mangrovibrevibacter* Zan *et al*. 2025, 13^3^	gen. nov.	*Mangrovibrevibacter kandeliae*	150	[90]
*Mangrovibrevibacter kandeliae* Zan *et al*. 2025, 13^3^	sp. nov.	CSK15Z-1 (=JCM 34923=MCCC 1K07183)	150	[90]
*Maribacter hydrothermalis* Lin *et al*. 2021, 2819	sp. nov.	T28 (=CGMCC 1.15788=JCM 31510)	123	[91]
*Marinifaba* Sui *et al*. 2021, 952	gen. nov.	*Marinifaba aquimaris*	47	[92]
*Marinifaba aquimaris* Sui *et al*. 2021, 952	sp. nov.	SM1970 (=KCTC 72844=MCCC 1K04323)	47	[92]
*Marinobacter arenosus* Lee *et al*. 2022, 5^3^	sp. nov.	CAU 1620 (=KCTC 82431=MCCC 1K06079)	65	[93]
*Marinomonas algarum* Xue *et al*. 2022, 10^3^	sp. nov.	E8 (=KCTC 92201=MCCC 1K07070)	74	[94]
*Marivita roseacus* Budinoff *et al*. 2011, 264	sp. nov.	CB1052 (=ATCC BAA 1914=DSM 23118)	168	[95]
*Martelella soudanensis* Lee *et al*. 2021, 10^3,56^	sp. nov.	NC18 (=KCTC 82174=NBRC 114661)^57^	137	[96]
*Megasphaera intestinihominis* Hitch *et al*. 2025, 14^3^	sp. nov.	CLA-AA-H81 (=DSM 118069=LMG 33597)	28	[22]
*Meridianimarinicoccus marinus* Kang *et al*. 2025, 7^3^	sp. nov.	DFM31 (=KEMB 021938=KCTC 8650=JCM 37289)	104	[97]
*Mesorhizobium marinum* Zan *et al*. 2025, 11^3,58^	sp. nov.	ZMM04-4 (=KCTC 8273=MCCC 1K08883)	149	[98]
*Micromonospora thermarum* Pansomsuay *et al*. 2023, 7^3^	sp. nov.	HSS6-12 (=JCM 17127=TBRC 17270)^59^	38	[99]
*Micromonospora zeae* Shen *et al*. 2014, 741	sp. nov.	NEAU-gq9 (=CGMCC 4.7092=DSM 45882)	172	[100]
*Microvirga arvi* Damdintogtokh *et al*. 2022, 5^3^	sp. nov.	BT689 (=KACC 22016=NBRC 114858)	69	[101]
*Microvirga jeongseonensis* Maeng *et al*. 2022, 3^3^	sp. nov.	BT688 (=KCTC 82701=NBRC 114857)	59	[102]
*Mucilaginibacter corticis* Akter and Huq 2020, 496	sp. nov.	MAH-19 (=CGMCC 1.13657=KACC 19745)	122	[103]
*Mucilaginibacter ginsenosidivorans* Kim *et al*. 2017, 1386	sp. nov.	Gsoil 3017 (=JCM 17081=KACC 14954)	21	[104]
*Natronomicrosphaera* Sorokin *et al*. 2025, 11^3^	gen. nov.	*Natronomicrosphaera hydrolytica*	134	[105]
*Natronomicrosphaera hydrolytica* Sorokin *et al*. 2025, 11^3^	sp. nov.	AB-hyl4 (=DSM 117994=UQM 41914)^60^	134	[105]
*Neopararhizobium* Hördt *et al*. 2020, 42^3^	gen. nov.	*Neopararhizobium haloflavum*	161	[106]
*Neopararhizobium haloflavum* (Shen *et al*. 2018) Hördt *et al*. 2020, 47^3^	comb. nov. [basonym: *Pararhizobium haloflavum* Shen *et al*. 2018]	XC0140 (=KCTC 52582=MCCC 1K03228)^61^	161	[106]
*Neorhizobium populisoli* (Shen *et al*. 2022) Ma *et al*. 2023, 12^3^	comb. nov. [basonym: *Rhizobium populisoli* Shen *et al*. 2022]	XQZ8 (=GDMCC 1.2201=JCM 34442)	157	[5]
*Nitratireductor mangrovi* Ye *et al*. 2020, 1337	sp. nov.	SY7 (=KCTC 72110=MCCC 1K03723)	92	[107]
*Nocardioides astragali* Xu *et al*. 2018, 1161	sp. nov.	HH06 (=CGMCC 4.7327=NBRC 112322)	32	[108]
*Nocardioides bruguierae* Chen *et al*. 2023, 9^3^	sp. nov.	BSK12Z-3 (=CGMCC 4.7709=JCM 34554)	151	[109]
*Nocardioides jiangxiensis* Wan *et al*. 2024, 7^3^	sp. nov.	WY-20 (=GDMCC 4.317=KACC 23379)	51	[110]
*Nocardioides xinjiangensis* Mo *et al*. 2025, 9^3^	sp. nov.	SYSU D00514 (=CGMCC 1.18622=KCTC 49488=MCCC 1K05001)	9	[111]
*Oceanospirillum sediminis* Zhao *et al*. 2022, 4^3^	sp. nov.	D5 (=KCTC 62987=MCCC 1K06061)	58	[112]
*Oenococcus sicerae* Cousin *et al*. 2019, 307	sp. nov.	UCMA15228 (CIRM-BIA2288=DSM 107163)	81	[113]
*Oryzicola* Chhetri *et al*. 2021, 1932	gen. nov.	*Oryzicola mucosus*	85	[114]
*Oryzicola mucosus* Chhetri *et al*. 2021, 1932	sp. nov.	ROOL2 (=KCTC 82711=NBRC 114717)	85	[114]
*Paenibacillus humicola* Lee *et al*. 2024, 6^3^	sp. nov.	TW40 (=KCTC 43469=NBRC 116016)	96	[115]
*Paenibacillus kandeliae* Zan *et al*. 2025, 9^3^	sp. nov.	JQZ6Y-1 (=CGMCC 1.18974=JCM 34547)	108	[116]
*Paenibacillus roseipurpureus* corrig. Hwang *et al*. 2025, 10^3, 62^	sp. nov.	MBLB1832 (=JCM 34221=KCTC 43262)	53	[117]
*Paenibacillus roseus* Akter *et al*. 2021, 4002	sp. nov.	MAHUQ-46 (=CGMCC 1.17353=KACC 21242)	125	[118]
*Paenibacillus tyrphae* corrig. Aw *et al*. 2016, 7^3,63^	sp. nov.	MSt1 (=DSM 100708=MCCC 1K01247)	34	[119]
*Paenilisteria* Bouznada *et al*. 2025, 22^3^	gen. nov.	*Paenilisteria rocourtiae*	26	[120]
*Paenilisteria newyorkensis* (Weller *et al*. 2015) Bouznada *et al*. 2025, 24^3^	comb. nov. [basonym: *Listeria newyorkensis* Weller *et al*. 2015]	FSL M6-0635 (=DSM 28861=NCTC 13937)^64^	26	[120]
*Paenilisteria rocourtiae* (Leclercq *et al*. 2010) Bouznada *et al*. 2025, 23^3^	comb. nov. [basonym: *Listeria rocourtiae* Leclercq *et al*. 2010]	Allerberger 700284/02 (=CECT 7972=DSM 22097)^65^	26	[120]
*Paenilisteria rustica* (Carlin *et al*. 2021) Bouznada *et al*. 2025, 24^3^	comb. nov. [basonym: *Listeria rustica* Carlin *et al*. 2021]	FSL W9-0585 (=CCUG 74665=LMG 31922)	26	[120]
*Paenirhizobium* Ma *et al*. 2023, 9^3^	gen. nov.	*Paenirhizobium daejeonense*	157	[5]
*Paenirhizobium daejeonense* (Quan *et al*. 2005) Ma *et al*. 2023, 10^3^	comb. nov. [basonym: *Rhizobium daejeonense* Quan *et al*. 2005]	L61 (=DSM 17795=JCM 21505)^66^	157	[5]
*Paenirhizobium naphthalenivorans* (Kaiya *et al*. 2018) Ma *et al*. 2023, 11^3^	comb. nov. [basonym: *Rhizobium naphthalenivorans* Kaiya *et al*. 2018]	TSY03b (=KCTC 23252=NBRC 107585)	157	[5]
*Paenirhizobium selenitireducens* (Hunter *et al*. 2008) Ma *et al*. 2023, 11^3^	comb. nov. [basonym: *Rhizobium selenitireducens* corrig. Hunter *et al*. 2008]	B1 (=ATCC BAA-1503=DSM 25018)^67^	157	[5]
*Pantoea trifolii* Wdowiak-Wróbel *et al*. 2024, 10^3^	sp. nov.	MMK2 (=DSM 115063=LMG 33049)	17	[121]
*Parabacteroides timonensis* Bilen *et al*. 2019, 8^3^	sp. nov.	Marseille-P3236 (=CCUG 71183=CSUR P3236)	138	[122]
*Paraburkholderia caffeinitolerans* Gao *et al*. 2016, 1480	sp. nov.	CF3 (=CGMCC 1.15105=LMG 28688)	23	[123]
*Parafrigoribacterium humi* OuYang *et al*. 2025, 6^3^	sp. nov.	SYSU BS000231 (=GDMCC 1.3816=KCTC 59001)	10	[124]
*Parafrigoribacterium soli* OuYang *et al*. 2025, 6^3^	sp. nov.	SYSU BS000078 (=GDMCC 1.4599=KCTC 59245)	10	[124]
*Pedobacter bambusae* Won *et al*. 2015, 570	sp. nov.	THG-G118 (=JCM 19364=KACC 17544)	82	[125]
*Pedobacter deserti* Huang *et al*. 2024, 8^3^	sp. nov.	SYSU D00382 (=CGMCC 1.18627=KCTC 82279=MCCC 1K04972)	12	[126]
*Pedobacter namyangjuensis* Kim *et al*. 2013, 29^68^	sp. nov.	5G38 (=KACC 13938=NBRC 107692)	173	[127]
*Peptoniphilus grossensis* Mishra *et al*. 2012, 329^69^	sp. nov.	ph5 (=CSUR P184=DSM 25475)	178	[128]
*Peptoniphilus obesi* Mishra *et al*. 2013, 367	sp. nov.	ph1 (=CSUR P187=DSM 25489)	176	[129]
*Peptoniphilus pacaensis* Diop *et al*. 2019, 14^3,70^	sp. nov.	Kh-D5 (=CSUR P2270=DSM 101839)	131	[130]
*Persephonella atlantica* François *et al*. 2021, 8^3^	sp. nov.	MO1340 (=JCM 34026=UBOCC M-3359	80	[131]
*Peteryoungia rhizophila* (Gao *et al*. 2020) Rahi *et al*. 2021, 3601	comb. nov. [basonym: *Rhizobium rhizophilum* Gao *et al*. 2020]^71^	7209–2 (=CGMCC 1.15691=DSM 103161)	159	[132]
*Peteryoungia wuzhouensis* (Yuan *et al*. 2018) Rahi *et al*. 2021, 3601	comb. nov. [basonym: *Rhizobium wuzhouense* Yuan *et al*. 2018]	W44 (=GDMCC 1.1257=KCTC 62194)^72^	159	[132]
*Phyllobacterium pellucidum* Park *et al*. 2021, 2650	sp. nov.	BT25 (=KCTC 62765=NBRC 114381)	84	[133]
*Pontibacter burrus* Park *et al*. 2021, 774	sp. nov.	BT327 (=KCTC 72412=NBRC 114376)	144	[134]
*Pontitalea* Yoon 2025, 5^3^	gen. nov.	*Pontitalea aquivivens*	139	[135]
*Pontitalea aquivivens* Yoon 2025, 5^3^	sp. nov.	KMU-169 (=KCCM 90598=NBRC 117093)	139	[135]
*Propionivibrio militaris* Thrash *et al*. 2010, 341^73^	sp. nov.	MP (=ATCC BAA-1728=DSM 21683)	170	[136]
*Pseudoflavonifractor intestinihominis* Hitch *et al*. 2025, 15^3^	sp. nov.	CLA-AP-H29 (=DSM 118073=LMG 33601)	28	[22]
*Pseudogemmobacter faecipullorum* Lin *et al*. 2022, 7^3^	sp. nov.	CC-YST710 (=BCRC 81286=JCM 34182)	162	[137]
*Pseudomonas hunanensis* Gao *et al*. 2014, 23	sp. nov.	LV (=CICC 10558=NCCB 100446)	174	[138]
*Pseudomonas imrae* Salvà-Serra *et al*. 2025, 10^3^	sp. nov.	16FHM2 (=CCUG 74779=CECT 30571)	5	[139]
*Pseudomonas pharyngis* Tohya *et al*. 2023, 3^3^	sp. nov.	BML-PP036 (=JCM 34583=LMG 32252)	128	[140]
*Pseudomonas sihuiensis* Wu *et al*. 2014, 786	sp. nov.	WM-2 (=CGMCC 1.12407=KCTC 32246)	167	[141]
*Pseudoramibacter faecis* Wang *et al*. 2025, 15^3^	sp. nov.	HA2172 (=CGMCC 1.18049=KCTC 25722)	7	[12]
*Rhizobium binxianense* Liu *et al*. 2025, 7^3^	sp. nov.	BJ04 (=CCTCC AB 2022367=JCM 35885)	147	[142]
*Rhizobium kunmingense* Shen *et al*. 2010, 147	sp. nov.	LXD30 (=CGMCC 1.8903=KCTC 22609)	175	[143]
*Robertmurraya yapensis* Hitch *et al*. 2025, 15^3^	sp. nov.	CLA-AA-H227 (=DSM 113004=LMG 33018)	28	[22]
*Roseateles cavernae* Zhang *et al*. 2025, 6^3^	sp. nov.	YIM B04394 (=CCTCC AB 2021502=KCTC 92438=NBRC 115775)	109	[144]
*Roseateles flavus* Woo *et al*. 2025, 10^3^	sp. nov.	2.12 (=KACC 23730=TBRC 19013)	110	[145]
*Roseateles hydrophilus* Woo *et al*. 2025, 11^3^	sp. nov.	UHG3 (=KACC 22636=TBRC 16347)	110	[145]
*Roseateles paludis* Woo *et al*. 2025, 11^3^	sp. nov.	DJS-2–20 (=KACC 23731=TBRC 19014)	110	[145]
*Rubrilithobacter* corrig. Lian *et al*. 2025, 8^3,74^	gen. nov.	*Rubrilithobacter danxiaensis* corrig.	8	[146]
*Rubrilithobacter danxiaensis* corrig. Lian *et al*. 2025, 8^3,68^	sp. nov.	SYSU DXS3258 (=KCTC 102322=MCCC 1K09420)	8	[146]
*Rufibacter psychrotolerans* Liu *et al*. 2024, 8^3^	sp. nov.	SYSU D00308 (=CGMCC 1.18621=KCTC 82275=MCCC 1K04970)	11	[147]
*Ruminococcoides intestinale* Hitch *et al*. 2025, 15^3^	sp. nov.	CLA-JM-H38 (=DSM 118486=LMG 33604)	28	[22]
*Ruminococcoides intestinihominis* Hitch *et al*. 2025, 16^3^	sp. nov.	CLA-AA-H171 (=DSM 114689=LMG 33587)	28	[22]
*Ruthenibacterium intestinale* Hitch *et al*. 2025, 16^3^	sp. nov.	CLA-JM-H11 (=DSM 114604=LMG 33032)	28	[22]
*Simplicispira hankyongi* Siddiqi *et al*. 2020, 336	sp. nov.	NY-02 (=LMG 31165=KACC 19731)	126	[148]
*Solibaculum intestinale* Hitch *et al*. 2025, 16^3^	sp. nov.	CLA-JM-H44 (=DSM 114601=LMG 33034)	28	[22]
*Sphingobium aromaticivastans* Nguyen and Kim 2019, 159	sp. nov.	UCM-25 (=DSM 105181=KACC 19288)	68	[149]
*Sphingomonas caeni* Liu *et al*. 2023, 692	sp. nov.	LB-2 (=GDMCC 1.3630=NBRC 116168)^75^	52	[150]
*Sphingomonas natans* Choi *et al*. 2024, 9^3^	sp. nov.	BIUV-7 (=KCTC 92961=NBRC 116546)	94	[151]
*Sphingomonas telluris* Siddiqi *et al*. 2023, 4^3^	sp. nov.	SM33 (=KACC 22222=LMG 32193)	35	[152]
*Spiribacter onubensis* León *et al*. 2025, 1^3^	sp. nov.	ag22IC6-221 (=CCM 9385=CECT 30947)	49	[153]
*Spirillospora tritici* Song *et al*. 2018, 1480	sp. nov.	SJ 21 (=CGMCC 4.7420=JCM 32390)	63	[154]
*Spirosoma aureum* Maeng *et al*. 2020, 2208	sp. nov.	BT328 (=KCTC 72365=NBRC 114506)	140	[155]
*Sporomusa intestinalis* Hattori *et al*. 2013, 323^76^	sp. nov.	Tc2A (=DSM 17189=JCM 13218)	164	[156]
*Staphylococcus ursi* Perreten *et al*. 2020, 4643	sp. nov.	MI 10–1553 (=ATCC TSD-55=CCOS 1900)	48	[157]
*Streptococcus taonis* Lee *et al*. 2024, 5^3^	sp. nov.	ST22-14 (=BCRC 81402=NBRC 116002)	57	[158]
*Streptococcus vaginalis* Chao *et al*. 2021, 5480	sp. nov.	P1L01 (=BCRC 81289=NBRC 114754)	56	[159]
*Streptomyces adonidis* Du *et al*. 2025, 10^3^	sp. nov.	NEAU-BLH26 (=JCM 36414=MCCC 1K08678)	93	[160]
*Streptomyces antimicrobicus* Chanama *et al*. 2023, 15^3^	sp. nov.	SMC277 (=NBRC 115422=TBRC 15568)	120	[161]
*Streptomyces colonosanans* Law *et al*. 2017, 11^3^	sp. nov.	MUSC 93J (=DSM 102042=MCCC 1K02298)	22	[162]
*Streptomyces odontomachi* Tunvongvinis *et al*. 2024, 734	sp. nov.	ODS25 (=NBRC 115862=TBRC 16204)	141	[163]
*Streptomyces qaidamensis* Zhang *et al*. 2018, 883	sp. nov.	S10 (=CGMCC 4.7315=JCM 31184)	90	[164]
*Streptomyces soli* Xing *et al*. 2020, 1691^77^	sp. nov.	LAM7114 (=CGMCC 4.7581=JCM 32822)	132	[165]
*Streptomyces tagetis* Chhetri *et al*. 2024, 12^3^	sp. nov.	RG38 (=KCTC 49624=TBRC 15113)	62	[166]
*Superficieibacter* Potter *et al*. 2018, 10^3^	gen. nov.	*Superficieibacter electus*	24	[167]
*Superficieibacter electus* Potter *et al*. 2018, 10^3^	sp. nov.	BP-1 (=ATCC BAA-2937=NBRC 113412)	24	[167]
*Tabrizicola caldifontis* Habib *et al*. 2025, 6^3^	sp. nov.	YIM 73028 (=CGMCC 1.16151=KCTC 52713)	13	[168]
*Taklimakanibacter albus* Tian *et al*. 2025, 7^3^	sp. nov.	YIM B02566 (=CGMCC 1.18656=JCM 34643)	111	[169]
*Terrimonas alba* Xiong *et al*. 2025, 8^3^	sp. nov.	R1 (=GDMCC 1.4673=JCM 37058=KACC 23743=KCTC 102293)	50	[170]
*Tessaracoccus massiliensis* Seck *et al*. 2016, 11	sp. nov.	SIT-7 (=CSUR P1301=DSM 29060)	97	[171]
*Thermococcus marinus* Jolivet *et al*. 2004, 225^78^	sp. nov.	EJ1 (=DSM 15227=JCM 11825)	165	[172]
*Thioclava kandeliae* Mou *et al*. 2025, 5^3^	sp. nov.	I07B-00821 (=CPCC 100088=KCTC 32191)	103	[173]
*Thiohalorhabdus methylotropha* Sorokin *et al*. 2025, 9^3,79^	sp. nov.	Cl-TMA (=JCM 35977=UQM 41915)	135	[174]
*Thiomonas arsenivorans* Battaglia-Brunet *et al*. 2006, 107^80^	sp. nov.	b6 (=DSM 16361=LMG 22795)	177	[175]
*Tullyiplasma* Gupta *et al*. 2019, 584	gen. nov.	*Tullyiplasma multilocale*	15	[176]
*Tullyiplasma multilocale* (Hill *et al*. 1992) Gupta *et al*. 2019, 584^81^	comb. nov. [basonym: *Acholeplasma multilocale* Hill *et al*. 1992]	PN525 (=ATCC 49900=DSM 116231)^82^	15	[176]
*Uliginosibacterium flavum* Kang *et al*. 2017, 598	sp. nov.	JJ3220 (=JCM 19465=KACC 17644)	67	[177]
*Uliginosibacterium silvisoli* Lv *et al*. 2025, 6^3^	sp. nov.	H3 (=KCTC 8564=MCCC 1K09133)^83^	106	[178]
*Umboniibacteraceae* Ho *et al*. 2025, 8^3^	fam. nov.	*Umboniibacter*	16	[179]
*Ureibacillus yapensis* (Yu *et al*. 2020) Yadav *et al*. 2024, 14^3,84^	comb. nov. [basonym: *Lysinibacillus yapensis* Yu *et al*. 2020]	ylb-03 (=JCM 32871=MCCC 1A12698)	41	[180]
*Ventrimonas* Hitch *et al*. 2025, 16^3^	gen. nov.	*Ventrimonas faecis*	28	[22]
*Ventrimonas faecis* Hitch *et al*. 2025, 16^3^	sp. nov.	CLA-AP-H27 (=DSM 118072=LMG 33600)	28	[22]
*Vibrio marinisediminis* Zhao *et al*. 2021, 813	sp. nov.	404 (=KCTC 62958=MCCC 1H00367)	95	[181]
*Waltera hominis* Hitch *et al*. 2025, 16^3^	sp. nov.	CLA-AA-H183 (=DSM 114684=LMG 33586)	28	[22]

For references to Validation Lists 1–71, see *Int J Syst Bacteriol* **49** (1999) 1325. Lists 72–197 were published in *Int J Syst Evol Microbiol* **50** (2000) 3, 423, 949, 1415, 1699, 1953; **51** (2001) 1, 263, 793, 1229, 1619, 1945; **52** (2002) 3, 685, 1075, 1437, 1915; **53** (2003) 1, 373, 627, 935, 1219, 1701; **54** (2004) 1, 307, 631, 1005, 1425, 1909; **55** (2005) 1, 547, 983, 1395, 1743, 2235; **56** (2006) 1, 499, 925, 1459, 2025, 2507; **57** (2007) 1, 433, 893, 1371, 1933, 2449; **58** (2008) 1, 529, 1057, 1511, 1993, 2471; **59** (2009) 1, 451, 923, 1555, 2129, 2647; **60** (2010) 1, 469, 1009, 1477, 1985, 2509; **61** (2011) 1, 475, 1011, 1499, 2025, 2563; **62** (2012) 1, 473, 1017, 1443, 2045, 2549; **63** (2013) 1, 797, 1577, 2365, 3131, 3931; **64** (2014) 1, 693, 1455, 2184, 3603; **65** (2015) 1, 741, 1112, 2017, 2777, 3767; **66** (2016) 1, 1603, 1913, 2463, 3761, 4299; **67** (2017) 1, 529, 1095, 2075, 3140, 4291; **68** (2018) 1, 693, 1411, 2130, 2707, 3379; **69** (2019) 5, 597, 1247, 1844, 2627, 3313; and **70** (2020) 1, 1443, 2960, 4043, 4844, 5596. Lists 197–224 were published in *Int J Syst Evol Microbiol* **71** (2021) 004600, 004688, 004773, 004846, 004943, 005096; **72** (2022) 005167, 005260, 005331, 005422, 005517, 005592; **73** (2023) 005709, 005812, 005845, 005931, 005997, 006080; **74** (2024) 006173, 006229, 006275, 006398, 006452, 006501; and **75** (2025) 006562, 006640, 006699, 006801.

1Abbreviations of culture collections cited in this list can be found at https://lpsn.dsmz.de/text/collections.

2Priority number assigned according to the date the documentation and request for validation are received.

3The journal in which the name was effective published does not have continuous pages.

4The species was named in honour of one of the co-authors, contravening Recommendation 6(7) of the ICNP.

5The effective publication states that the type strain was also deposited as TRM 40139 and CCTCC AA 2012020, but no documentation was supplied.

6The effective publication states that the type strain was also deposited as MTCC 11562, but no documentation was supplied.

7*Allorhizobium pseudoryzae* (Zhang *et al*. 2011) Mousavi *et al.* 2016 is a homotypic synonym.

8The effective publication states that the type strain was also deposited as ACCC 10380 and DSM 26483, but no documentation was supplied.

9The effective publication states that the type strain was also deposited as ACCC 05916 and DSM 19478, but no documentation was supplied.

10The list editors have corrected *diazotrophicus* to *diazotrophicum* (di.a.zo.tro’phi.cum. … N.L. neut. adj. *diazotrophicum*, …).

11The list editors have corrected *nitritogenes* to *nitritigenes* (ni.tri.ti’ge.nes. N.L. masc. n. *nitris*, nitrite; Gr. v. *gennao*, produce, engender; N.L. masc. part. adj. *nitritigenes*, nitrite-producing).

12The protologue heading must state gen. nov. instead of gen. Nov., and the preferred syllabification and etymology are as follows: An.ae.ro.ru’dis. Gr. pref. *an*, not; Gr. masc. n. aer, *air*; L. fem. n. *rudis*, a small stick; N.L. fem. n. *Anaerorudis*, anaerobic small stick.

13The protologue heading must state sp. nov. instead of sp. nov, and the preferred syllabification and etymology are as follows: cel.lu.lo.si.vo’rans. N.L. neut. n. *cellulosum*, cellulose; L. pres. part. *vorans*, devouring; N.L. part. adj. *cellulosivorans*, cellulose devouring).

14The preferred etymology is N.L. neut. n. *ginsenosidum*, ginsenoside; L. pres. part. *mutans*, transforming, converting; N.L. part adj. *ginsenosidimutans*, ginsenoside-converting.

15The nomenclature reviewers have corrected the epither to *dokdonensis* (dok.do.nen’sis. N.L. masc. adj. *dokdonensis*, named after Dokdo …).

16The effective publication gives DSM 11806 instead of DSM 118068.

17The effective publication states that the type strain was also deposited as DSM 106029, but no documentation was supplied.

18The effective publication states that the type strain was also deposited as DSM 106020, but no documentation was supplied.

19The list editors have corrected *metallicus* to *metallicum* (me.tal’li.cum. L. neut. n. *metallicum*, of or belonging to metal, referring to the fact that the bacterium is resistant to various heavy metals).

20The effective publication states that the type strain was also deposited as CCBAU 101093 and LMG 8673, but no documentation was supplied.

21The list editors have corrected *vitisensis* to *vitis* (vi’tis. L. gen. n. *vitis*, of *Vitis*, the generic name of the grapevine).

22The culture collection numbers are found in the abstract, but not in the protologue.

23The list editors have corrected *histidini* to *histidinae* (his.ti.di‘nae. N.L. gen. n. *histidinae*, of histidine).

24The list editors have corrected *indicus* to *indicum* (in’di.cum. L. neut. adj. *indicum*, Indian).

25The preferred etymology is N.L. neut. adj. *amazonitimonense*, referring to the Amazonian forest where the stool sample was collected and La Timone hospital where the strain was isolated.

26The etymology must state N.L. neut. adj. instead of L. masc. adj.

27The effective publication states that the type strain was also deposited as CGMCC 1.60052, but no documentation was supplied.

28The preferred syllabification and etymology are ae.gi.ce.ra’tis. … of the genus *Aegiceras*.

29The list editors have corrected *haemomassiliense* to *haematomassilense* (hae.ma.to.mas.si.li.en’se. Gr. neut. n. *haima*, blood; L. masc. adj. *massiliensis*, pertaining to Marseille; N.L. neut. adj. *haematomassiliense*, of blood from Marseille, where the type strain was isolated.

30The effective publication states that the type strain was also deposited as CGMCC 1.7045, but no documentation was supplied. Culture collection numbers are given in the abstract, not in the protologue.

31The preferred syllabification and etymology are lim.nae’us. Gr. masc. adj. …

32The protologue heading has *D. dismutans* instead of *Desulfovibrio dismutans*. The preferred etymology is Gr. adv. *dis*, … L. pres. part. *mutans*, … N.L. part. adj. *dismutans*, …

33The effective publication states that the type strain was also deposited as UNIQEM U934, but no documentation was supplied.

34The effective publication states that the type strain was also deposited as KACC 19437, but no documentation was supplied.

35The effective publication states that the type strain was also deposited as ACCC 60381, but no documentation was supplied.

36The preferred etymology is Gr. neut. n. *edaphos*, …

37The preferred etymology is N.L. fem. n. *cremea*, cream-coloured.

38The effective publication states that the type strain was also deposited as CGMCC 1.5857, but no documentation was supplied.

39The name is an earlier homotypic synonym of *Tullyiplasma multilocale* (Hill *et al*. 1992) Gupta *et al*. 2019.

40The genus name was corrected to *Flaviaestuariibacter* in accordance with Judicial Opinion 118 [182].

41The preferred etymology is N.L. fem. adj. instead of L. adj.

42Culture collection numbers are given in the abstract, not in the protologue.

43The type species was not explicitly mentioned in the text.

44The preferred syllabification and etymology are se.di.men’ti. L. gen. n. *sedimenti*, of sediment, …

45The effective publication states that the type strain was also deposited as KACC 13775, but no documentation was supplied.

46The effective publication states that the type strain was also deposited as KCTC 59021, but no documentation was supplied.

47The preferred etymology is N.L. fem. n. instead of L. fem. n.

48The list editors have corrected *fonsfrigidae* to *frigidifontis* (fri.gi.di.fon’tis. L. masc. adj. *frigidus*, cold; L. masc. n. *fons*, spring, seep; N.L. gen. n. *frigidifontis*, of a cold seep).

49The preferred etymology is N.L. gen. fem. n. instead of N.L. gen. fem. N.

50The preferred etymology is L. fem. n. *sphaera* instead of N.L. fem. n. *sphaera*.

51The preferred etymology is: Gr. fem. n. *limne*, lake; Gr. masc. adj. *philos*, loving; N.L. fem. adj. *limnophila*, lake loving.

52The effective publication states that the type strain was also deposited as KCTC 39963, but no documentation was supplied.

53The list editors have corrected *tyrosinelyticus* to *tyrosinilyticus* (ty.ro.si.ni.ly’ti.cus. N.L. fem. n. *tyrosina*, tyrosine; N.L. masc. adj. *lyticus* (from Gr. masc. adj. *lytikos*), dissolving; N.L. masc. adj. *tyrosinilyticus*, decomposing tyrosine).

54The etymology must state N.L. fem. adj. instead of N.L. masc. adj.

55The effective publication states that the type strain was also deposited as FBCC-B1, but no documentation was supplied.

56The preferred etymology is N.L. fem. adj. instead of N.L. masc./fem. adj.

57In the abstract, KCTC 82174 was given as KTCT 82174.

58The preferred etymology is L. neut. adj. instead of L. neut. Adj.

59The effective publication states that the type strain was also deposited as BCC 41915, but no documentation was supplied.

60The protologue gives DSMZ 117994 instead of DSM 117994.

61The strain number XC0140 was not mentioned in the effective publication of the new combination, but was given in the description of the basonym.

62The list editors have corrected *roseopurpureus* to *roseipurpureus* (ro.se.i.pur.pu’re.us. L. masc. adj. *roseus*, red; L. masc. adj. *purpureus*, purple; N.L. masc. adj. *roseipurpureus*, red-purple).

63The list editors have corrected *tyrfis* to *tyrphae* (tyr’phae. Modern Gr. fem. n. *tyrphe*, peat; N.L. gen. n. *tyrphae*, of peat).

64The effective publication states that the type strain was also deposited as LMG 28310, but no documentation was supplied.

65The effective publication states that the type strain was also deposited as CIP 109804, but no documentation was supplied.

66The effective publication states that the type strain was also deposited as CCBAU 10050, IAM 15042, KCTC 12121 and NBRC 102495, but no documentation was supplied.

67The effective publication states that the type strain was also deposited as LMG 24075 and NRRL B-41997, but no documentation was supplied.

68The etymology must state N.L. masc. adj. instead of N.L. fem. adj.

69The preferred etymology is gros.sen’sis. L. masc. adj. *grossus*, thick; N.L. masc. adj. *grossensis*, intended to mean ‘of an obese patient’.

70The etymology must state N.L. masc. adj. instead of N.L. gen. masc. n.

71The effective publication gave *Rhizobium rhizophila*.

72The effective publication states that the type strain was also deposited as CCTCC AB 2017179, but no documentation was supplied.

73The preferred syllabification and etymology are mi.li.ta’ris. L. masc. adj. *militaris*, war-like …

74The list editors have corrected *Rubrolithibacter* to *Rubrilithobacter* (Ru.bri.li.tho.bac’ter. L. masc. adj. *ruber*, red; Gr. masc. n. *lithos*, stone, rock; N.L. masc. n. *bacter*, rod; N.L. masc. n. *Rubrilithobacter*, red rock-dwelling rod).

75The effective publication states that the type strain was also deposited as NBRC 115,102, but no documentation was supplied.

76The preferred syllabification and etymology are in.tes.ti.na’lis. L. neut. n. *intestinum*, intestine; N.L. fem. adj. *intestinalis*, intestinal, pertaining to the intestine.

77Later homotypic synonym of *Actinacidiphila soli* Madhaiyan *et al.* 2022.

78The protologue heading must state *Thermococcus marinus* instead of *T. marinus*. The preferred syllabification and etymology are ma.ri’nus. L. masc. adj. *marinus*, of the sea.

79The abstract gives *methyotrophus* instead of *methylotropha*.

80The preferred syllabification and etymology are ar.se.ni.vo’rans. N.L. neut. n. *arsenum* (from Gr. masc. adj. *arsen*, virile, masculine), arsenic; L. pres. part. *vorans*, devouring; N.L. part. adj. *arsenivorans*, arsenic eating).

81Later homotypic synonym of *Entomoplasma multilocale* (Hill *et al.* 1992) Gasparich and Kuo 2019.

82The effective publication states that the type strain was also deposited as NCTC 11723, but no documentation was supplied.

83The effective publication states that the type strain was also deposited as JSACC 33374, but no documentation was supplied.

84The preferred etymology is N.L. masc. adj. instead of N.L. masc./fem. adj.

## Corrigenda

In validation list no. 223 [183], the basonym of *Hyphococcus luteus* nom. nov. Ye *et al*. 2025 is *Marinicaulis flavus* Yu *et al*. 2018 and not *M. flavus* Yu *et al*. 2019 as listed.

Validation list no. 223 [183] includes validation of the names *Roseiconus* Kumar *et al*. 2021, *Roseiconus lacunae* Kumar *et al*. 2021 and *Roseiconus nitratireducens* Kumar *et al*. 2021. The type strains of the two species were isolated in India, and therefore, permission from a third party is needed to access the strains. Therefore, the names *Roseiconus* Kumar *et al*. 2021, *Roseiconus lacunae* Kumar *et al*. 2021 and *Roseiconus nitratireducens* Kumar *et al*. 2021 must be considered effectively but not validly published.

## Abbreviations

ICNP: International Code of Nomenclature of Prokaryotes
*IJSEM*: *International Journal of Systematic and Evolutionary Microbiology*

## References

[1] Oren A, Arahal DR, Göker M, Moore ERB, Rossello-Mora R (2023). International Code of Nomenclature of Prokaryotes. Prokaryotic Code (2022 Revision). Int J Syst Evol Microbiol.

[2] He J, Hong L, Song M, Zhang Y, Zhang W (2025). Corrigendum to “Diverse *Acinetobacter* species and plasmid-driven spread of carbapenem resistance in pharmaceutical settings in China” [Environ. Int. 198 (2025) 109373]. Environ Int.

[3] Saker R, Bouras N, Meklat A, Holtz MD, Klenk H-P (2022). Genome-based reclassification of *Actinopolyspora righensis* Meklat *et al*. 2013 as a later heterotypic synonym of *Actinopolyspora lacussalsi* Guan *et al*. 2013 and description of *Actinopolyspora lacussalsi* subsp. *lacussalsi* subsp. nov. and *Actinopolyspora lacussalsi* subsp. *righensis* subsp. nov. Arch Microbiol.

[4] Ajmi N, Duman M, Ay H, Saticioglu IB (2025). Genomic and pangenomic insights into *Aeromonas salmonicida* subsp. *oncorhynchi* subsp. nov. *Pathogens*.

[5] Ma T, Xue H, Piao C, Jiang N, Li Y (2023). Phylogenomic reappraisal of the family *Rhizobiaceae* at the genus and species levels, including the description of *Ectorhizobium quercum* gen. nov., sp. nov. Front Microbiol.

[6] Ndongo S, Armstrong N, Raoult D, Fournier P-E (2022). Reclassification of eight *Akkermansia muciniphila* strains and description of *Akkermansia massiliensis* sp. nov. and *Candidatus Akkermansia* timonensis, isolated from human feces. Sci Rep.

[7] Mousavi SA, Willems A, Nesme X, de Lajudie P, Lindström K (2015). Revised phylogeny of *Rhizobiaceae*: Proposal of the delineation of *Pararhizobium* gen. nov., and 13 new species combinations. Syst Appl Microbiol.

[8] Ahn S, Lee Y, Weerawongwiwat V, Kim J-H, Lee J-S (2025). *Altererythrobacter arenosus* sp. nov., isolated from marine sediment. Curr Microbiol.

[9] Lee J-C, Whang K-S (2021). *Altererythrobacter segetis* sp. nov., isolated from farmland soil. Curr Microbiol.

[10] Luo B, Su J-Y, Zhang Y-F, Xiao Y-H, Peng Y-L (2024). *Alteromonas arenosi* sp. nov., a novel bioflocculant-producing bacterium, isolated from intertidal sand. Antonie van Leeuwenhoek.

[11] Bing RG Metabolic engineering and optimization of non-native solventogenic lignocellulose fermentation by the extreme thermophile *Anaerocellum bescii*.

[12] Wang X-M, Huang H-J, Sun X-W, Wei R-Q, Chen H-Y (2025). Identification and characterization of two novel members of the family *Eubacteriaceae*, *Anaerofustis butyriciformans* sp. nov. and *Pseudoramibacter faecis* sp. nov., isolated from human feces. Microorganisms.

[13] Zhang R, Zeng Y, Wang T-Q, Wang X-M, Shi Y-Y (2020). *Anaerophilus nitritogenes* gen. nov., sp. nov., isolated from salt lake sediment in Xinjiang Province, China. Antonie van Leeuwenhoek.

[14] El Houari A, Carpenter M, Chaplin D, Golyshin P, McDonald JE (2025). Taxonomic description and genome sequence of *Anaerorudis cellulosivorans* gen. *Nov*. sp. nov., a novel cellulose- and Xylan-degrading bacterium of the *Bacteroidota* phylum isolated from a lab-scale methanogenic landfill bioreactor digesting municipal solid waste. Syst Appl Microbiol.

[15] Jia C, Cui H-C, Han Y-Q, Fu T-Y, Du R (2018). *Ancylomarina psychrotolerans* sp. nov., isolated from sediments of Fildes Peninsula and emended the description of genus *Ancylomarina*. Antonie van Leeuwenhoek.

[16] Shi W, Takano T, Liu S (2012). *Anditalea andensis* gen. nov., sp. nov., an alkaliphilic, halotolerant bacterium isolated from extreme alkali-saline soil. Antonie van Leeuwenhoek.

[17] Siddiqi MZ, Shafi SM, Im W-T (2017). Complete genome sequencing of *Arachidicoccus ginsenosidimutans* sp. nov., and its application for production of minor ginsenosides by finding a novel ginsenoside-transforming β-glucosidase. RSC Adv.

[18] Koh H-W, Kang M-S, Lee K-E, Lee E-Y, Kim H (2019). *Arthrobacter dokdonellae* sp. nov., isolated from a plant of the genus *Campanula*. J Microbiol.

[19] Chen L, Wang S-C, Ma C-H, Zheng D-X, Du Z-J (2018). *Ascidiaceibacter salegens* gen. nov., sp. nov., isolated from an ascidian. Antonie van Leeuwenhoek.

[20] Li K, Ye Z, Chen G, Zheng K, Yin J (2023). *Atopomonas sediminilitoris* sp. nov., isolated from beach sediment of Zhairuo Island, China. Antonie van Leeuwenhoek.

[21] Cadoret F, Alou MT, Afouda P, Traore IS, Bréchard L (2017). Noncontiguous finished genome sequences and description of *Bacillus massiliglaciei*, *Bacillus mediterraneensis*, *Bacillus massi-linigeriensis*, *Bacillus phocaeensis* and *Bacillus tuaregi*, five new species identified by culturomics. New Microbe and New Infect.

[22] Hitch TCA, Masson JM, Pauvert C, Bosch J, Nüchtern S (2025). HiBC: a publicly available collection of bacterial strains isolated from the human gut. Nat Commun.

[23] Modesto M, Satti M, Watanabe K, Scarafile D, Huang C-H (2025). Erratum to “Characterization of two novel species of the genus *Bifidobacterium*: *Bifidobacterium saimiriisciurei* sp. nov. and *Bifidobacterium platyrrhinorum* sp. nov.” [Syst. Appl. Microbiol. 43 (2020) 126111]. Syst Appl Microbiol.

[24] Román-Ponce B, Li YH, Vásquez-Murrieta MS, Sui XH, Chen WF (2015). *Brevibacterium metallicus* sp. nov., an endophytic bacterium isolated from roots of *Prosopis laegivata* grown at the edge of a mine tailing in Mexico. Arch Microbiol.

[25] Jiang L, Jeon D, Kim J, Lee CW, Peng Y (2021). Pyomelanin-producing *Brevundimonas vitisensis* sp. nov., isolated from grape (*Vitis vinifera* L.). Front Microbiol.

[26] Zhang M-D, Zhou Z-Y, Kou Y-Y, Lu D-C, Du Z-J (2023). *Brumimicrobium oceani* sp. nov., isolated from coastal sediment lake and environmental adaptability analysis. Antonie van Leeuwenhoek.

[27] Ong KS, Aw YK, Lee LH, Yule CM, Cheow YL (2016). *Burkholderia paludis* sp. nov., an antibiotic-siderophore producing novel *Burkholderia cepacia* complex species, isolated from Malaysian tropical peat swamp soil. Front Microbiol.

[28] Xu Y, Chen L, Yang L, Pan L, Zheng M (2025). *Chitinibacter mangrovi* sp. nov., a novel chitin-degrading bacterium isolated from mangrove sediment. Curr Microbiol.

[29] Liu X, Liang H, Zhu Y, Zhou Q, Shao S (2025). *Chitinophaga defluvii* sp. nov., a cyhalofop-butyl-degrading bacterium isolated from municipal sludge. Curr Microbiol.

[30] Kim M, Cha I-T, Lee K-E, Park S-J (2024). *Chryseobacterium gotjawalense* sp. nov. isolated from soil in the volcanic forest Gotjawal, Jeju Island. Curr Microbiol.

[31] Chhetri G, Kim I, Kim J, So Y, Seo T (2022). *Chryseobacterium tagetis* sp. nov., a plant growth promoting bacterium with an antimicrobial activity isolated from the roots of medicinal plant (*Tagetes patula*). J Antibiot.

[32] Lee SA, Kim Y, Sang M-K, Song J, Kwon S-W (2019). *Chryseolinea soli* sp. nov., isolated from soil. J Microbiol.

[33] Octaviana S, Lorenczyk S, Ackert F, Fenske L, Wink J (2022). Four new members of the family *Cytophagaceae*: *Cryseosolibacter histidini* gen. nov., sp. nov., *Chryseosolibacter indicus* gen. nov., *Dawidia cretensis*, gen. nov., sp. nov., and *Dawidia soli*, gen. nov., sp. nov. isolated from diverse habitat. Antonie van Leeuwenhoek.

[34] Alou MT, Ndongo S, Frégère L, Labas N, Andrieu C (2018). Taxono-genomic description of four new *Clostridium* species isolated from human gut: ‘*Clostridium amazonitimonense*’, ‘*Clostridium merdae*’, ‘*Clostridium massilidielmoense*’ and ‘*Clostrdium nigeriense*’. New Microbe and New Infect.

[35] Wang J-Y, Pan W-Y, Yang X-Y, Wang Z-Z, Su Y (2023). *Clostridium brassicae* sp. nov., a strictly anaerobic bacterium isolated from high-salt industrial wastewater. Curr Microbiol.

[36] Hosny M, Abdallah RA, Bou Khalil J, Fontanini A, Baptiste E (2019). *Clostridium pacaense*: a new species within the genus *Clostridium*. New Microbe and New Infect.

[37] Xiang M-X, Miao C-P, Zhang D-Y, Wang J, Li Y-Q (2024). Description and genomic characterization of *Cohnella caldifontis* sp. nov., isolated from hot springs in Yunnan province, south-west China. Antonie van Leeuwenhoek.

[38] Luo Y, Zan C, Chen M, Liu F, Chen X (2025). *Consotaella aegiceratis* sp. nov., a novel endophytic bacterium isolated from root of *Aegiceras corniculatum*. Curr Microbiol.

[39] Boxberger M, Antezack A, Magnien S, Cassir N, La Scola B (2020). Draft genome and description of *Corynebacterium haemomassiliense* strain Marseille-Q3615T sp. nov., a new bacterium isolated from a 59-year-old man with chronic obstructive pulmonary disease symptoms. New Microbe and New Infect.

[40] Shin S, Kahng H-Y (2017). *Cyclobacterium sediminis* sp. nov. isolated from a sea cucumber aquaculture farm and emended description of the genus *Cyclobacterium*. J Microbiol.

[41] Shi H, Rao MPN, Chen Q-Q, Quadri SR, Li W-J (2025). Description of *Cytobacillus mangrovibacter* sp. nov., and *Cytobacillus spartinae* sp. nov., isolated from mangrove sediment. Curr Microbiol.

[42] Lee Y, Kim J-H, Yoon J-H, Lee J-S, Sukhoom A (2023). Description of *Defluviimonas salinarum* sp. nov. with the potential of benzene-degradation isolated from saltern in the Yellow Seacoast. FEMS Microbiol Lett.

[43] Sun J, Shen P, Chao H, Wu B (2009). Isolation and identification of a new radiation-resistant bacterium *Deinococcus guangxiensis* sp. nov. and analysis of its radioresistant character. Acta Microbiol Sin.

[44] Gundlapally SR, Garcia-Pichel F (2017). Description of *Deinococcus oregonensis* sp. nov., from biological soil crusts in the Southwestern arid lands of the United States of America. Arch Microbiol.

[45] Watanabe M, Takahashi A, Kojima H, Miyata N, Fukui M (2022). *Desulfofustis limnaeus* sp. nov., a freshwater sulfate-reducing bacterium isolated from marsh soil. Arch Microbiol.

[46] Sorokin DY, Merkel AY, Ziganshin RH, Kublanov IV (2025). Growth physiology, genomics, and proteomics of *Desulfurivibrio dismutans* sp. nov., an obligately chemolithoautotrophic, sulfur disproportionating and ammonifying haloalkaliphile from soda lakes. Front Microbiol.

[47] Sorokin DY, Merkel AY (2022). Bergey’s Manual of Systematics of Archaea and Bacteria.

[48] Sorokin DY, Merkel AY (2022). Bergey’s Manual of Systematics of Archaea and Bacteria.

[49] Sorokin DY, Merkel AY (2022). Bergey’s Manual of Systematics of Archaea and Bacteria.

[50] Qiu S, Hu H, Zhuo Y, Su Y, Yang Y (2025). *Devosia alba* sp. nov., a potential nitrite-reducing bacterium isolated from a mudflat. Curr Microbiol.

[51] Park S, Won S-M, Yoon J-H (2018). *Dokdonia ponticola* sp. nov., a carrageenan-degrading bacterium of the family *Flavobacteriaceae* isolated from seawater. Curr Microbiol.

[52] Chen L, Gao X, Ma Q, Liu H, Wang X (2020). *Dyadobacter luteus* sp. nov., isolated from rose rhizosphere soil. Arch Microbiol.

[53] Baek J, Weerawongwiwat V, Kim J-H, Yoon J-H, Lee J-S (2021). *Echinicola arenosa* sp. nov., isolated from marine sand. Arch Microbiol.

[54] Xue J-Y, Zhang M-Y, Zhang Y, Cheng J, Liu L-C (2019). *Edaphovirga cremea* gen. nov., sp. nov., isolated from the rhizospheric soil of *Codonopsis clematidea*. J Microbiol.

[55] Gasparich GE, Kuo C-H (2019). Genome analysis-based union of the genus *Mesoplasma* with the genus *Entomoplasma*. Int J Syst Evol Microbiol.

[56] Guo H-B, Zhu H-T, Zhang Y-F, Yang F, Ma X-T (2025). *Erwinia aeris* sp. nov., a novel bacterium isolated from the surface of an ore in Hubei Province, China. Curr Microbiol.

[57] Zheng G, Chen Z-G, Jiang R-J, Yang Z-J (2015). *Feifantangia zhejiang-ensis* gen. nov., sp. nov., a marine bacterium isolated from seawater of the East China Sea. Antonie van Leeuwenhoek.

[58] Nou NO, Covington JK, Lai D, Mayali X, Seymour CO (2025). Author Correction: Genome-guided isolation of the hyperthermophilic aerobe Fervidibacter sacchari reveals conserved polysaccharide metabolism in the Armatimonadota. Nat Commun.

[59] Wang H-L, Zhang J, Sun L (2018). *Fictibacillus iocasae* sp. nov., isolated from the deep-sea sediment in Pacmanus, Manus Basin. Arch Microbiol.

[60] Yoon J, Yasumoto-Hirose M, Kasai H (2025). *Flagellimonas algarum* sp. nov., isolated from dense mats of filamentous algae. Folia Microbiol.

[61] Jang JH, Maeng SH, Jung HY, Kim MK, Subramani G (2020). *Flaviaesturariibacter flavus* sp. nov., isolated from soil in Jeju Island. Arch Microbiol.

[62] Maeng S, Park Y, Lee SE, Han JH, Bai J (2021). *Flavisolibacter longurius* sp. nov., isolated from soil. Arch Microbiol.

[63] Yang L-L, Xin Y-H, Liu Q (2025). *Flavobacterium algoriphilum* sp. nov., *Flavobacterium arabinosi* sp. nov., *Flavobacterium cryoconiti* sp. nov., *Flavobacterium galactosi* sp. nov., *Flavobacterium melibiosi* sp. nov., and *Flavobacterium algoris* sp. nov., six novel cold-adapted bacteria isolated from glaciers. BMC Microbiol.

[64] Kim S-R, Kim Y-J, Nguyen N-L, Min J-W, Jeon J-N (2011). *Flavobacterium ginsengiterrae* sp. nov., isolated from a ginseng field. J Gen Appl Microbiol.

[65] Yang F, Yue J, Wang Q, Zha W, Zhou Y (2025). *Frateuria hangzhou-ensis* sp. nov. isolated from soil of moso bamboo forest in Hangzhou, China. Curr Microbiol.

[66] Zhang W-Y, Yuan Y, Su D-Q, He X-P, Han S-B (2018). *Gallaecimonas mangrovi* sp. nov., a novel bacterium isolated from mangrove sediment. Antonie van Leeuwenhoek.

[67] Ryu SW, Moon JC, Oh BS, Yu SY, Bak JE (2025). *Gallibacterium faecale* sp. nov., isolated from dairy cow feces. Curr Microbiol.

[68] Jayan JN, Srinivasan S, Lee S-S (2025). Isolation and characterisation of *Gracilimonas aurantiaca* sp. nov., a novel bacterium isolated from tidal-flat sediment. Curr Microbiol.

[69] Liu J-Q, Zhu L-R, Mao Y-L, Ma X, Hou J (2025). Genome-based reclassification of two *Haloarcula* species and characterization of *Haloarcula montana* sp. nov. Biology.

[70] Anani H, Tidjani Alou M, Fontanini A, Raoult D, Lagier J-C (2020). Taxono-genomics and description of *Haloimpatiens massiliensis* sp. nov., a new bacterium isolated from the gut of a healthy infant. New Microbe and New Infect.

[71] Jeong J, Weerawongwiwat V, Lee Y, Kim J-H, Sukhoom A (2022). *Halomarinibacterium sedimenti* gen. nov., sp. nov., a carotenoid pigment-producing bacterium isolated from marine sediment. Arch Microbiol.

[72] Zhao H, Wang C, Tian Y, Zhang Y, Wu Y (2020). *Halomonas marinisediminis* sp. nov., a moderately halophilic bacterium isolated from the Bohai Gulf. Curr Microbiol.

[73] Wang L, Shao Z (2021). Aerobic denitrification and heterotrophic sulfur oxidation in the genus *Halomonas* revealed by six novel species characterizations and genome-based analysis. Front Microbiol.

[74] Park JK, Chang D-H, Rhee M-S, Jeong H, Song J (2021). *Heminiphilus faecis* gen. nov., sp. nov., a member of the family *Muribaculaceae*, isolated from mouse faeces and emended description of the genus *Muribaculum*. Antonie van Leeuwenhoek.

[75] Castelli M, Petroni G (2025). An evolutionary-focused review of the *Holosporales* (*Alphaproteobacteria*): diversity, host interactions, and taxonomic re-ranking as *Holosporineae* subord. nov. Microb Ecol.

[76] Damdintogtokh T, Cha I-T, Kim MK (2021). *Hymenobacter guriensis* sp. nov., and *Hymenobacter duratus* sp. nov., radiation-resistant species isolated from soil in South Korea. Curr Microbiol.

[77] Oh H, Lee HB, Kim MK (2022). *Hymenobacter lucidus* sp. nov., and *Hymenobacter nitidus* sp. nov., isolated from soil. Arch Microbiol.

[78] Li X, Li C, Lai Q, Li G, Sun F (2016). *Hyphomonas pacifica* sp. nov., isolated from deep sea of the Pacific Ocean. Antonie van Leeuwenhoek.

[79] Feng L-Y, Zhao J-Y, Shi Z-F, Li M-G, Pu T (2025). *Intrasporangium zincisolvens* sp. nov., a novel actinobacterium isolated from rhizosphere soil. Curr Microbiol.

[80] Zhang J, Zhang Y, Liu R, Cai R, Liu F (2021). *Iocasia fonsfrigidae* NS-1 gen. nov., sp. nov., a novel deep-sea bacterium possessing diverse carbohydrate metabolic pathways. Front Microbiol.

[81] Zhang Y, Liu F, Li F-N, Chen M-S, Ma X (2022). *Jiella avicennia*e sp. nov., a novel endophytic bacterium isolated from bark of *Avicennia marina*. Arch Microbiol.

[82] Suenari Y, Matsubara T, Hiroshima Y, Pahlevi MR, Shimada H (2025). Characterization of *Lachnoanaerobaculum sanguinis* sp. nov., isolated from a blood culture of an acute myeloid leukemia patient with chemotherapy-related bacteremia. Anaerobe.

[83] Rast P, Glöckner I, Boedeker C, Jeske O, Wiegand S (2017). Three novel species with peptidoglycan cell wall from the new genus *Lacunisphaera* gen. nov. in the family Opitutaceae of the verrucomicrobial subdivision 4. Front Microbiol.

[84] Cai Y, Tao W-Z, Ma Y-J, Cheng J, Zhang M-Y (2018). *Leifsonia flava* sp. nov., a novel actinobacterium isolated from the rhizosphere of *Aquilegia viridiflora*. J Microbiol.

[85] Aja JA, Llorin LD, Lim KRQ, Teodosio JJ, Sioson EJ (2025). Genome mining reveals the biosynthetic potential of a novel *Lysinibacillus zambalensis* sp. nov., isolated from a hyperalkaline spring. *Arch Microbiol*.

[86] Du J, Singh H, Ngo HTT, Won K, Kim K-Y (2015). *Lysobacter tyrosine-lyticus* sp. nov. isolated from Gyeryongsan national park soil. J Microbiol.

[87] Schiffer CJ, Ehrmann MA (2025). *Macrococcus capreoli* sp. nov., a new fosfomycin resistant species isolated from feces and nasal swabs of deer. Syst Appl Microbiol.

[88] Mašlaňová I, Kovařovic V, Botka T, Švec P, Sedláček I (2025). Evidence of *in vitro mecB*-mediated β-lactam antibiotic resistance transfer to *Staphylococcus aureus* from *Macrococcus psychroto-lerans* sp. nov., a psychrophilic bacterium from food-producing animals and human clinical specimens. Appl Environ Microbiol.

[89] Baek K, Choi A (2023). *Macromonas nakdongensis* sp. nov., isolated from freshwater and characterization of bacteriophage BK-30P-the first phage that infects genus *Macromonas*. Microorganisms.

[90] Zan C, Zheng Z, Chen X, Chen M, Li F (2025). Description and genomic characterization of *Mangrovibrevibacter kandeliae* gen. nov. sp. nov., a novel carotenoid-producing endophytic bacterium isolated from branch of mangrove plants. Antonie van Leeuwenhoek.

[91] Lin H, Lin D, Zhang M, Ye J (2021). *Maribacter hydrothermalis* sp. nov., isolated from shallow-sea hydrothermal systems off Kueishantao Island. Curr Microbiol.

[92] Sui X, He X-Y, Liu N-H, Dang Y-R, Cha Q-Q (2021). *Marinifaba aquimaris* gen. nov., sp. nov., a novel chitin-degrading gammaproteobacterium in the family *Alteromonadaceae* isolated from seawater of the Mariana Trench. Antonie van Leeuwenhoek.

[93] Lee Y, Weerawongwiwat V, Kim J-H, Suh MK, Kim HS (2022). *Marino-bacter arenosus* sp. nov., a halotolerant bacterium isolated from a tidal flat. Arch Microbiol.

[94] Xue J-H, Zhang B-N, Zhang F, Liu Y-Y, Wu W-J (2022). Comparative genomic analysis of the genus *Marinomonas* and taxonomic study of *Marinomonas algarum* sp. nov., isolated from red algae *Gelidium amansii*. Arch Microbiol.

[95] R. Budinoff C, R. Dunlap J, Hadden M, Buchan A (2011). *Marivita roseacus* sp. nov., of the family *Rhodobacteraceae*, isolated from a temperate estuary and an emended description of the genus *Marivita*. J Gen Appl Microbiol.

[96] Lee J-Y, Lee D-H, Kim D-H (2021). Characterization of *Martelella soudanensis* sp. nov., isolated from a mine sediment. Microorganisms.

[97] Kang M-S, Yu J-Y, Kim H-S, Dong K, Srinivasan S (2025). *Meridianimarinicoccus marinus* sp. nov., isolated from tidal flat. Curr Microbiol.

[98] Zan C, Ma X, Zheng Z, Li F, Yang X (2025). Description and genomic characterization of *Mesorhizobium marinum* sp. nov., a bacterium isolated from sea sediment. Syst Appl Microbiol.

[99] Pansomsuay R, Duangupama T, Pittayakhajonwut P, Intaraudom C, Suriyachadkun C (2023). *Micromonospora thermarum* sp. nov., an actinobacterium isolated from hot spring soil. Arch Microbiol.

[100] Shen Y, Zhang Y, Liu C, Wang X, Zhao J (2014). *Micromonospora zeae* sp. nov., a novel endophytic actinomycete isolated from corn root (*Zea mays* L.). J Antibiot.

[101] Damdintogtokh T, Park Y, Maeng S, Oh HJ, Bang M (2022). *Microvirga terrestris* sp. nov., and *Microvirga arvi* sp. nov., isolated from soil in South Korea. Arch Microbiol.

[102] Maeng S, Park Y, Oh H, Bang M, Baigalmaa J (2022). *Microvirga jeongseonensis* sp. nov., isolated from soil in South Korea. Arch Microbiol.

[103] Akter S, Huq MdA (2020). *Mucilaginibacter corticis* sp. nov., isolated from bark of *Pinus koraiensis*. Antonie van Leeuwenhoek.

[104] Kim MM, Siddiqi MZ, Im W-T (2017). *Mucilaginibacter ginsenosidivorans* sp. nov., isolated from soil of ginseng field. Curr Microbiol.

[105] Sorokin DY, Merkel AY, Bale NJ, Koenen M, Sinninghe Damsté JS (2025). *Natronomicrosphaera hydrolytica*, gen. nov., sp. nov., a first representative of the phylum *Planctomycetota* from soda lakes. Syst Appl Microbiol.

[106] Hördt A, López MG, Meier-Kolthoff JP, Schleuning M, Weinhold L-M (2020). Analysis of 1,000+ type-strain genomes substantially improves taxonomic classification of *Alphaproteobacteria*. Front Microbiol.

[107] Ye Y, Yan C, Nie Y, Zhang J, Zhao Z (2020). *Nitratireductor mangrovi* sp. nov., a nitrate reducing bacterium isolated from mangrove soil. Curr Microbiol.

[108] Xu L, Zhang Y, Li C, Wang X, Liu J (2018). *Nocardioides astragali* sp. nov., isolated from a nodule of wild *Astragalus chrysopterus* in northwestern China. Antonie van Leeuwenhoek.

[109] Chen X, Zheng Z, Li F, Ma X, Chen F (2023). Description and genomic characterization of *Nocardioides bruguierae* sp. nov., isolated from *Bruguiera gymnorhiza*. Syst Appl Microbiol.

[110] Wan Y, Sun T, Huang G, Tu B, Huang M (2024). *Nocardioides jiangxi-ensis* sp. nov., a novel actinobacterium isolated from lakeside soil, exhibiting the biosynthesis potential of mycofactocin. Antonie van Leeuwenhoek.

[111] Mo Y-J, Liu J, Huang J, Zheng Z-H, Li S (2025). *Nocardioides xinjiang-ensis* sp. nov., a novel species isolated from desert soil. Antonie van Leeuwenhoek.

[112] Zhao H, Tian Y, Sun X, Wu Q, Chen S (2022). *Oceanospirillum sediminis* sp. nov., isolated from coastal sediment in the Yellow Sea. Curr Microbiol.

[113] Cousin FJ, Le Guellec R, Chagnot C, Goux D, Dalmasso M (2019). *Oenococcus sicerae* sp. nov., isolated from French cider. Syst Appl Microbiol.

[114] Chhetri G, Kim J, Kim I, Kang M, So Y (2021). *Oryzicola mucosus* gen. nov., sp. nov., a novel slime producing bacterium belonging to the family *Phyllobacteriaceae* isolated from the rhizosphere of rice plants. Antonie van Leeuwenhoek.

[115] Lee H, Chaudhary DK, Lim OB, Kim D-U (2024). *Paenibacillus silvisoli* sp. nov. and *Paenibacillus humicola* sp. nov., isolated from forest soil. Arch Microbiol.

[116] Zan C-S, Ma X, Chen X-H, Chen M-S, Tuo L (2025). Proposal of *Paenibacillus kandeliae* sp. nov., isolataed from leaf of *Kandelia candel*. Curr Microbiol.

[117] Hwang CY, Cho E-S, Nam Y-D, Park S-L, Lim S-I (2025). A novel carotenoid-producing bacterium, *Paenibacillus roseopurpureus* sp. nov., isolated from Korean marine mud. Antonie van Leeuwenhoek.

[118] Akter S, Wang X, Lee S-Y, Rahman MM, Park J-H (2021). *Paenibacillus roseus* sp. nov., a ginsenoside-transforming bacterium isolated from forest soil. Arch Microbiol.

[119] Aw Y-K, Ong K-S, Lee L-H, Cheow Y-L, Yule CM (2016). Newly isolated *Paenibacillus tyrfis* sp. nov., from Malaysian tropical peat swamp soil with broad spectrum antimicrobial activity. Front Microbiol.

[120] Bouznada K, Belaouni HA, Saker R, Chaabane Chaouch F, Meklat A (2025). Phylogenomic analyses of the *Listeriaceae* family support species reclassification and proposal of a new family and new genera. Antonie van Leeuwenhoek.

[121] Wdowiak-Wróbel S, Kalita M, Palusińska-Szysz M, Marek-Kozaczuk M, Sokołowski W (2024). *Pantoea trifolii* sp. nov., a novel bacterium isolated from *Trifolium rubens* root nodules. Sci Rep.

[122] Bilen M, Mbogning Fonkou MD, Khelaifia S, Tomei E, Cadoret F (2019). Taxonogenomics description of *Parabacteroides timonensis* sp. nov. isolated from a human stool sample. Microbiol Open.

[123] Gao Z-Q, Zhao D-Y, Xu L, Zhao R-T, Chen M (2016). *Paraburkholderia caffeinitolerans* sp. nov., a caffeine degrading species isolated from a tea plantation soil sample. Antonie van Leeuwenhoek.

[124] OuYang Y-T, Chen L-B, Jiao J-Y, Hu Z-X, Wang J-S (2025). *Parafrigori-bacterium soli* sp. nov. and *Parafrigoribacterium humi* sp. nov., two novel siderophore-synthesizing species isolated from black soil. Syst Appl Microbiol.

[125] Won KH, Kook M, Yi T-H (2015). *Pedobacter bambusae* sp. nov., isolated from soil of a bamboo plantation. Antonie van Leeuwenhoek.

[126] Huang J, Li S, She T-T, Liu J, Mo Y-J (2024). *Pedobacter deserti* sp. nov., a novel species isolated from desert soil. Antonie van Leeuwenhoek.

[127] Kim D-U, Kim Y-J, Shin D-H, Weon H-Y, Kwon S-W (2013). *Pedobacter namyangjuensis* sp. nov. isolated from soil and reclassification of *Nubsella zeaxanthinifaciens* Asker *et al*. 2008 as *Pedobacter zeaxanthinifaciens* comb. nov. J Microbiol.

[128] Mishra AK, Hugon P, Robert C, Raoult D, Fournier P-E (2012). Non contiguous-finished genome sequence and description of *Peptoni-philus grossensis* sp. nov. Stand Genomic Sci.

[129] Mishra AK, Hugon P, Lagier J-C, Nguyen T-T, Robert C (2013). Non contiguous-finished genome sequence and description of *Peptoniphilus obesi* sp. nov. Stand Genomic Sci.

[130] Diop K, Diop A, Michelle C, Richez M, Rathored J (2019). Description of three new *Peptoniphilus* species cultured in the vaginal fluid of a woman diagnosed with bacterial vaginosis: *Peptoniphilus pacaensis* sp. nov., *Peptoniphilus raoultii* sp. nov., and *Peptoniphilus vaginalis* sp. nov. MicrobiologyOpen.

[131] François DX, Godfroy A, Mathien C, Aubé J, Cathalot C (2021). *Persephonella atlantica* sp. nov.: How to adapt to physico-chemical gradients in high temperature hydrothermal habitats. Syst Appl Microbiol.

[132] Rahi P, Khairnar M, Hagir A, Narayan A, Jain KR (2021). *Peter-youngia* gen. nov. with four new species combinations and description of *Peteryoungia desertarenae* sp. nov., and taxonomic revision of the genus *Ciceribacter* based on phylogenomics of *Rhizobiaceae*. Arch Microbiol.

[133] Park Y, Ten LN, Maeng S, Chang Y, Jung H-Y (2021). *Phyllobacterium pellucidum* sp. nov., isolated from soil. Arch Microbiol.

[134] Park Y, Maeng S, Han JH, Lee SE, Chang Y (2021). *Pontibacter fetidus* sp. nov. and *Pontibacter burrus* sp. nov., isolated from the soil. Arch Microbiol.

[135] Yoon J (2025). Polyphasic taxonomic analysis of *Pontitalea aqui-vivens* gen. nov., sp. nov., isolated from seawater. Curr Microbiol.

[136] Thrash JC, Pollock J, Torok T, Coates JD (2010). Description of the novel perchlorate-reducing bacteria *Dechlorobacter hydrogenophilus* gen. nov., sp. nov. and *Propionivibrio militaris*, sp. nov. Appl Microbiol Biotechnol.

[137] Lin S-Y, Hameed A, Tsai C-F, Tang Y-S, Young C-C (2022). Description of *Pseudogemmobacter faecipullorum* sp. nov., isolated from poultry manure. FEMS Microbiol Lett.

[138] Gao J, Li B, Wang H, Liu Z (2014). *Pseudomonas hunanensis* sp. nov., isolated from soil subjected to long-term manganese pollution. Curr Microbiol.

[139] Salvà-Serra F, Nimje P, Piñeiro-Iglesias B, Alarcón LA, Cardew S (2025). Description of *Pseudomonas imrae* sp. nov., carrying a novel class C β-lactamase gene variant, isolated from gut samples of Atlantic mackerel (*Scomber scombrus*). Front Microbiol.

[140] Tohya M, Teramoto K, Watanabe S, Hishinuma T, Shimojima M (2023). Correction for Tohya et al., “Whole-genome sequencing-based re-identification of *Pseudomonas putida*/*fluorescens* clinical isolates identified by biochemical bacterial identification systems”. Microbiol Spectr.

[141] Wu M, Wen J, Chang M, Yang G, Zhou S (2014). *Pseudomonas sihuiensis* sp. nov., isolated from a forest soil in South China. Antonie van Leeuwenhoek.

[142] Liu Y, Liu L, Wang Z, Huang X, Dong Y (2025). *Rhizobium binxianense* sp. nov. and *Rhizobium mulingense* sp. nov., isolated from nodules of *Phaseolus vulgaris* in Heilongjiang Province of China. Syst Appl Microbiol.

[143] Shen L, Zheng L-P, Liu H, Liu R, Zhang K-Y (2010). *Rhizobium kunmingense* sp. nov., isolated from rhizosphere soil of *Camptotheca acuminata* Decne. J Gen Appl Microbiol.

[144] Zhang Y-M, Jiang Q, Li M-Y, Miao Q-Y, Li Y-Q (2025). *Roseateles cavernae* sp. nov., a bacterium isolated from freshwater of the Old Huanglong Cave in Yunnan Province, South-West China. Curr Microbiol.

[145] Woo H, Chhetri G, Kim I, Park S, Lee H (2025). Three novel bacterial species, *Roseateles flavus* sp. nov., *Roseateles paludis* sp. nov., and *Roseateles hydrophilus* sp. nov., isolated from various water-related environments. Curr Microbiol.

[146] Lian W-H, He H-H, Wang Y, Zheng Z-H, Chen F (2025). *Rubrolithibacter danxiaensis* gen. nov., sp. nov. isolated from red sandy conglomerate in the Danxia Mountain. Antonie van Leeuwenhoek.

[147] Liu J, Li S, She T-T, Huang J, Lian W-H (2024). *Rufibacter psychrotolerans* sp. nov., a cold-tolerating novel species isolated from desert soil. Curr Microbiol.

[148] Siddiqi MZ, Sok W, Choi G, Kim SY, Wee J-H (2020). *Simplicispira hankyongi* sp. nov., a novel denitrifying bacterium isolated from sludge. Antonie van Leeuwenhoek.

[149] Nguyen TM, Kim J (2019). *Sphingobium aromaticivastans* sp. nov., a novel aniline- and benzene-degrading, and antimicrobial compound producing bacterium. Arch Microbiol.

[150] Liu B, Wan Y, Chen E, Huang M, Chen X (2023). *Sphingomonas caeni* sp. nov., a phenolic acid-degrading bacterium isolated from activated sludge. Antonie van Leeuwenhoek.

[151] Choi I, Srinivasan S, Kim MK (2024). *Sphingomonas Immobilis* sp. nov., and *Sphingomonas natans* sp. nov. bacteria isolated from soil. Arch Microbiol.

[152] Siddiqi MZ, Rajivgandhi G, Faiq M, Im W-T (2023). Isolation and characterization of *Sphingomonas telluris*, *Sphingomonas caseinilyticus* isolated from wet land soil. Curr Microbiol.

[153] León MJ, Vera-Gargallo B, de la Haba RR, Sánchez-Porro C, Ventosa A (2025). Author Correction: Integrating genomic evidence for an updated taxonomy of the bacterial genus *Spiribacter*. Sci Rep.

[154] Song J, Wang J, Sun T, Li C, He H (2018). *Spirillospora tritici* sp. nov., a novel actinomycete isolated from rhizosphere soil of *Triticum aestivum* L. Curr Microbiol.

[155] Maeng S, Park Y, Han JH, Lee SE, Zhang J (2020). *Spirosoma aureum* sp. nov., and *Hymenobacter russus* sp. nov., radiation-resistant bacteria in *Cytophagales* order isolated from soil. Antonie van Leeuwenhoek.

[156] Hattori S, Hongoh Y, Itoh T, Deevong P, Trakulnaleamsai S (2013). *Sporomusa intestinalis* sp. nov., a homoacetogenic bacterium isolated from the gut of a higher termite, *Termes comis* (Termitinae). J Gen Appl Microbiol.

[157] Perreten V, Kania SA, Bemis D (2020). *Staphylococcus ursi* sp. nov., a new member of the ‘*Staphylococcus intermedius* group’ isolated from healthy black bears. Int J Syst Evol Microbiol.

[158] Lee C-Y, Chan C-K, Chida M, Miyashita M, Lee Y-S (2024). *Streptococcus taonis* sp. nov., a novel bacterial species isolated from a blood culture of a patient. Arch Microbiol.

[159] Chao A-S, Lin C-Y, Chao A, Lee Y-S, Chang Y-C (2021). *Streptococcus vaginalis* sp. nov., a novel bacterial species isolated from vaginal swabs of a pregnant woman with diabetes. Arch Microbiol.

[160] Du R, Bing H, Lu W, Gao C, Kong F (2025). *Streptomyces adonidis* sp. nov., a novel actinomycete strain with antifungal activity isolated from the root of *Adonis amurensis* Regel. Antonie van Leeuwenhoek.

[161] Chanama M, Suriyachadkun C, Chanama S (2023). *Streptomyces antimicrobicus* sp. nov., a novel clay soil-derived actinobacterium producing antimicrobials against drug-resistant bacteria. PLoS One.

[162] Law JW-F, Ser H-L, Duangjai A, Saokaew S, Bukhari SI (2017). *Streptomyces colonosanans* sp. nov., a novel actinobacterium isolated from Malaysia mangrove soil exhibiting antioxidative activity and cytotoxic potential against human colon cancer cell lines. Front Microbiol.

[163] Tunvongvinis T, Jaitrong W, Suriyachadkun C, Sripreechasak P, Tanasupawat S (2024). *Streptomyces odontomachi* sp. nov., a novel actinobacterium with antimicrobial potential isolated from ants (*Odonto-machus simillimus* Smith, 1858). J Antibiot.

[164] Zhang B, Tang S, Chen X, Zhang G, Zhang W (2018). *Streptomyces qaidamensis* sp. nov., isolated from sand in the Qaidam Basin, China. J Antibiot.

[165] Xing J, Jiang X, Kong D, Zhou Y, Li M (2020). *Streptomyces soli* sp. nov., isolated from birch forest soil. Arch Microbiol.

[166] Chhetri G, Kim MJ, Kim I, Tran DVH, Kim Y-W (2024). *Streptomyces tagetis* sp. nov., a chromomycin producing bacteria isolated from the roots of *Tagetes patula*. Front Microbiol.

[167] Potter RF, D’Souza AW, Wallace MA, Shupe A, Patel S (2018). *Superficieibacter electus* gen. nov., sp. nov., an extended-spectrum β-lactamase possessing member of the *Enterobacteriaceae* family, isolated from intensive care unit surfaces. Front Microbiol.

[168] Habib N, Khan IU, Saqib M, Hejazi MS, Tarhriz V (2025). *Tabrizicola caldifontis* sp. nov., isolated from hot spring sediment sample. Curr Microbiol.

[169] Tian J-Y, Zhang Z, Zhao Y, Zhi X-Y, Yang L-L (2025). *Taklimakanibacter albus* sp. nov., an endophytic bacterial species isolated from the root of *Paris polyphylla* var. *yunnanensis*. Curr Microbiol.

[170] Xiong M, Wu H, Li J, Wan Y, Chen X (2025). *Terrimonas alba* sp. nov., an ochratoxin A degrading strain isolated from lakeside soil. Curr Microbiol.

[171] Seck E, Traore SI, Khelaifia S, Beye M, Michelle C (2016). *Tessaracoccus massiliensis* sp. nov., a new bacterial species isolated from the human gut. New Microbe and New Infect.

[172] Jolivet E, Corre E, L’Haridon S, Forterre P, Prieur D (2004). *Thermococcus marinus* sp. nov. and *Thermococcus radiotolerans* sp. nov., two hyperthermophilic archaea from deep-sea hydrothermal vents that resist ionizing radiation. Extremophiles.

[173] Mou T, Su J, Zhang J, Yu L-Y, Chen H-H (2025). *Thioclava kandeliae* sp. nov., Isolated from the rhizosphere soil of mangrove plant *Kandelia candel*. Curr Microbiol.

[174] Sorokin DY, Merkel AY, Gebbie W, Kalyuzhnaya MG (2025). *Thiohalorhabdus methylotropha* sp. nov., an extremely halophilic autotrophic methylotiotroph from hypersaline lakes. Syst Appl Microbiol.

[175] Battaglia-Brunet F, Joulian C, Garrido F, Dictor M-C, Morin D (2006). Oxidation of arsenite by *Thiomonas* strains and characterization of *Thiomonas arsenivorans* sp. nov. Antonie van Leeuwenhoek.

[176] Gupta RS, Son J, Oren A (2019). A phylogenomic and molecular markers based taxonomic framework for members of the order *Entomoplasmatales*: proposal for an emended order *Mycoplasmatales* containing the family *Spiroplasmataceae* and emended family *Mycoplasmataceae* comprised of six genera. Antonie van Leeuwenhoek.

[177] Kang JY, Chun J, Jahng KY (2017). *Uliginosibacterium flavum* sp. nov. isolated from an artificial lake. J Microbiol.

[178] Lv Y, Zhang Q, Liu L, He J, Wang S (2025). *Uliginosibacterium silvisoli* sp. nov., isolated from subtropical forest soil in Hunan Province, China. Curr Microbiol.

[179] Ho JY, Koh XQ, Kang DY, Low A, Hu D (2025). Discovery of a phylogenetically novel tropical marine *Gammaproteobacteria* elucidated from assembled genomes and the proposed transfer of the genus *Umboniibacter* from the family *Cellvibrionaceae* to *Umboniibacteraceae* fam. nov. Front Microbiol.

[180] Yadav A, Teware R, Bhatt A, Bhavsar Y, Maurya A (2024). *Ureibacillus aquaedulcis* sp. nov., isolated from freshwater well and reclassification of *Lysinibacillus yapensis* and *Lysinibacillus antri* as *Ureibacillus yapensis* comb. nov. and *Ureibacillus antri* comb. Nov. Arch Microbiol.

[181] Zhao H, Shan J, Wang T, Tian Y, Shen Y (2021). *Vibrio marinisediminis* sp. nov., isolated from marine sediment. Curr Microbiol.

[182] Arahal DR, Busse H-J, Bull CT, Christensen H, Chuvochina M (2022). Judicial opinions 112-122. Int J Syst Evol Microbiol.

[183] Oren A, Göker M (2025). Validation list no. 223. list of new names and new combinations effectively, but not validly, published. Int J Syst Evol Microbiol.

